# Plant-microbe interactions: PGPM as microbial inoculants/biofertilizers for sustaining crop productivity and soil fertility

**DOI:** 10.1016/j.crmicr.2024.100333

**Published:** 2024-12-16

**Authors:** Bibek Laishram, Okram Ricky Devi, Rinjumoni Dutta, T. Senthilkumar, Girish Goyal, Dinesh Kumar Paliwal, Narinder Panotra, Akhtar Rasool

**Affiliations:** a Department of Agronomy, Assam Agricultural University, Jorhat 785013, Assam, India; b Krishi Vigyan Kendra, TNAU, Dharmapuri, Tamil Nadu, India; c Acharya Narendra Deva University of Agriculture and Technology, Kumarganj, Ayodhya, India; d AICRP on Cotton, RVSKVV, BM College of Agriculture Khandwa, India; e Institute of Biotechnology, SKUAST Jammu, Jammu and Kashmir 180009, India; f Research Center for Chemistry - National Research and Innovation Agency (BRIN), KST BJ Habibie, Building 452, Setu, Tangerang Selatan 15314, Indonesia; g Department of Biotechnology, Manav Rachna International Institute of Research and Studies, Faridabad, Haryana, India

**Keywords:** Plant-microbiome interaction, PGPM, Microbial inoculants, Biofertilizers, Productivity, Soil health, Sustainable agriculture

## Abstract

Plant-microbe interactions play pivotal roles in sustaining crop productivity and soil fertility, offering promising avenues for sustainable agricultural practices. This review paper explores the multifaceted interactions between plants and various microorganisms, highlighting their significance in enhancing crop productivity, combating pathogens, and promoting soil health. Understanding these interactions is crucial for harnessing their potential in agricultural systems to address challenges such as food security and environmental sustainability. Therefore, the introduction of beneficial microbes into agricultural ecosystems by bio-augmentation reduces the negative effects of intensive, non-sustainable agriculture on the environment, society, and economy, into the mechanisms underlying the application of plant growth promoting microbes as microbial inoculants/biofertilizers; their interactions, the factors influencing their dynamics, and the implications for agricultural practices, emerging technologies and strategies that leverage plant-microbe interactions for improving crop yields, soil fertility, and overall agricultural sustainability.

## Highlights

• Plant growth-promoting microorganisms (PGPM) as microbial inoculants and biofertilizers.
• PGPM mechanisms in enhancing plant growth, nutrient uptake, and soil health.
• The interactions between plants and PGPMs, emphasizing their role in crop productivity.
• PGPM-mediated stress resistance and soil health improvement.

## 1 Introduction

Sustainable agricultural methods are essential in the face of growing environmental pressures, both biotic and abiotic, that have an impact on plant productivity worldwide (Khan and Lazali, 2023). For the goal to assure food security, agricultural output must be intensified in order to attain higher crop yields and overall production due to the world population's rapid expansion (FAO, 2017). However, due to the overuse of synthetic chemical pesticides and fertilizers, which worsen environmental degradation and pose health hazards to humans (Babin et al., 2019), agriculture is one of the human activities that most strongly contributes to the rise in chemical pollutants. On the other hand, the implications of climate change on interactions between plant pathogens are complex, with the ability to modify pathogen biology, host development, and disease severity. These changes have the potential to increase or decrease the incidence of diseases. Crop yields are negatively influenced by climate change-related issues like flooding, droughts, and extremely high temperatures. Low soil moisture levels and changes in plant physiology have been brought about by heat waves, severe droughts, and a shortage of water. Crops must have increased nutritional value, be resistant to disease, and be able to withstand stress from heavy metals, salt, and drought in order to achieve sustainable agriculture.

Microbes, which can be parasitic or mutualistic organisms, colonize the plant's above- and below-ground parts (Nadarajah and Rahman, 2021). These microbes may affect plants' growth and well-being in a beneficial, neutral, or detrimental way (Smith and Goodman, 1999; Berg et al., 2005). Microorganisms in the soil play a crucial role in protecting plants from stress by regulating phytohormones and improving the absorption of nutrients, among other processes that promote crop development and yield (Tkacz and Poole, 2015). Moreover, by inducing systemic resistance mechanisms in plants, these microbes increase their resilience to biotic stressors. The microbial interaction with plants acts as a catalyst in agriculture, enhancing output on its own (Nelson, 2004; Bhattacharyya et al., 2016). Plant growth-promoting microorganisms (PGPMs), particularly bacteria and fungi, present a practical means of achieving these goals (Rasool et al., 2021). These microorganisms can enhance a plant's nutrient-absorption capacity (Larsen et al., 2015) and water-use efficiency (Armada et al., 2014), as well as foster resistance against plant diseases (Turner et al., 2013; Kumar and Verma, 2018). Plant-microbe interactions are an achievable path towards agricultural sustainability since they are essential to maintaining soil fertility and crop productivity (Oldroyd, 2013). Long-term soil fertility and agricultural crop yield depend on interactions between plants and microbes (Parniske, 2008). Nevertheless, there are a lot of intricate relationships that are formed in the soil, especially in the rhizosphere, between the microorganisms, the crop, and the environment (Agrahari et al., 2020).

In order to enhance farming techniques, beneficial microorganisms can be added to the soil or inoculated. To improve crop health and yield and lessen the harmful effects of agrochemicals, microbial inoculants are administered into the soil or plants. It is an effective substitute for chemical treatment and may stabilize soil structure, manage diseases and pests, and encourage plant growth (Pandey et al., 2019). These inputs can be used as biopesticides, biocontrol agents, bioherbicides, and biofertilizers (Nadarajah and Rahman, 2021 and Rasool et al., 2024). This study examines the complex processes and modes of action underlying these interactions, as well as their bidirectional relationship and the particular areas in which they take place. In instance, beneficial bacteria play a variety of roles that are vital to the growth and well-being of plants. Since these PGPMs have both positive and antagonistic features that affect crop productivity and health, it is imperative to understand their method of action, the function of PGPMs in preserving soil fertility is investigated, focusing their contributions to the cycling of nutrients, the enhancement of soil structure, and general soil health. This work underscores how crucial it is to use plant-microbe interactions to address the problems of contemporary agriculture through a thorough investigation of these subjects.

## 2 Plant-Microbiome interactions

"Plant-microbe interaction" alludes to the different relationships that plants have with microorganisms in their local environment (Parniske, 2008). These interactions involve a complex network of relationships between various microorganisms, such as bacteria, fungi, viruses, and plants. These microorganisms can have both beneficial and detrimental effects (Reynolds et al., 2003) on the growth and health of plants, depending on the exact interactions that occur. Numerous microorganisms coexist in the environment of plants, including bacteria, oomycetes, fungi, archaea, and an as-yet-unexplored class of viruses (Agler et al., 2016; Swanson et al., 2009). The intricate, multilateral interactions between the biotic occupants of the abiotic environment and themselves form the composition of the plant microbiome. Microbes are classified as mutualistic, commensal, or pathogenic based on how a relationship affects the host.

### 2.1 Do plants interact with microbe?

**Fig. 1:**
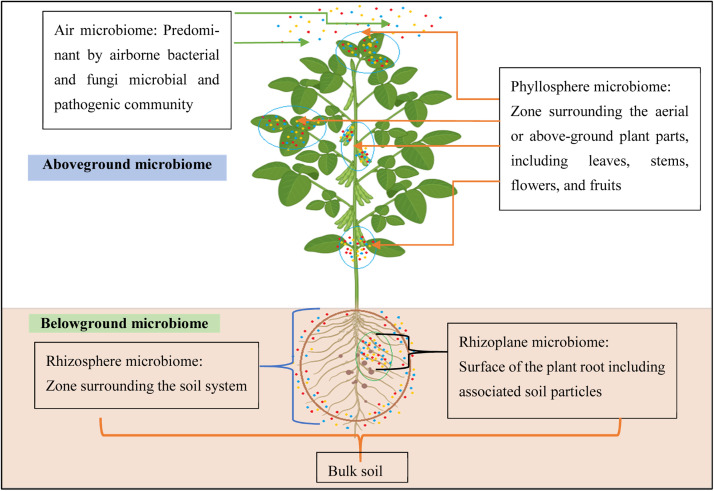
Regions of Plant-Microbiome interactions in connection with various microbiota that inhabit distinct niches on and inside plant tissue (*i.e*., above and below the microbiome) (Gopal and Gupta, 2019). In this section, the phyllosphere of microorganisms—which includes stems, leaves, seeds, flowers, and fruits—and the air microbiome—which includes bacteria and pathogens that are carried by the air—are the above-ground microbiome. The rhizosphere, a thin zone encircling the soil with the most active region of that frontier where biogeochemical processes impact a host of microbiomes, and the rhizoplane microbiome, which surrounds the surface of plant roots, make up the below-ground microbiome.

Plants and microbes interact in a variety of ways that could be advantageous or detrimental (Vandenkoornhuyse et al., 2015). The microbiome of plants is a mostly unexplored source of beneficial bacteria with a diversity of features and the unsuspected ability to govern plant development and success under difficult situations, claim Poudel et al. (2019). Microbiome are a group of microorganisms that reside in a certain habitat, including bacteria, fungi and viruses. In plants, a variety of microorganisms, both above and below ground (Fig. 1), coexist in dense populations for mutualistic purposes (Gopal and Gupta, 2016). These microbes can be classified as endophytes, or microorganisms that colonize inside plants and the majority of endophytes spread systemically in different plant compartments through the xylem (Compant et al., 2011); and epiphytes, which are organisms that colonize outside of plants, depending on the environments in which they do so (Brown et al., 2020). The ecology of endosphere and episphere microbiome is quite different, despite the fact that above-ground plant tissues (such as vegetative foliar sections, floral parts, and leaves) offer different homes to a wide variety of species containing bacteria, fungi, algae, archaea, and viruses. The phyllosphere microbiome, are often impacted by their surroundings i.e. the air microbiome and can either be disease-causing by pathogens or commensal (Nadarajah and Rahman, 2021).

The phyllosphere is a harsh and unstable environment that has several characteristics of oligotrophy, including dietary limitation in carbon and nitrogen. A multitude of variables related to various physicochemical and biotic constraints appear to be important for microbial adaptation to the phyllosphere environment, including exposure to air, water, soil, animal, or insect-borne microorganisms (Sivakumar et al., 2020; Frank et al., 2017).

The most common of them are the rhizosphere inhabitants, which are found in the regions nearest to the root system (Sivakumar et al., 2020). The rhizosphere is thought to be the most active among them, having a major influence on the development and nutritional condition of plants (Bakker et al., 2012). The small area of soil that is directly surrounded by roots and is impacted by both bacteria and root secretions is known as the rhizosphere. According to Panke-Buisse et al. (2015) and Bandyopadhyay et al. (2016), the subterranean system is primarily made up of soil, primary roots, lateral developments, and root hairs. These components determine how these elements interact with the vast array of microbial diversity in the rhizosphere, which in turn affects the plant's growth stages and resistance to various stresses. According to Philippot et al. (2013), the plant–root microbiome is the whole system in which plant roots interact with the rhizomicrobiome.

## 3 Rhizosphere interactions

Plants-microbes interact intricately in the rhizosphere, a dynamic region laden with myriad microorganisms and invertebrates, encompassing root-induced physical, chemical, and biological processes (Zhang et al. 2022). This sphere includes, various zones such as the rhizoplane, ectorhizosphere, and endorhizosphere (Morgan et al., 2005). The vital ecological balance in the rhizosphere hinges on root exudates, which, when provided, reward microbes with robust energy and nutrient sources. In return, these microorganisms stimulate further root exudation, demonstrating a symbiotic relationship. Exudates and mucilages from roots play a key role as recruiting agents, intricately regulating microbial interactions and gene expression (Egamberdieva et al., 2017; Compant et al., 2020). The process of root exudation enriches the rhizosphere with a plethora of primary metabolites—such as carbohydrates, amino acids, and organic acids—far more abundantly than secondary metabolites like phenolics, flavonoids, glucosinolates, and auxins (Badri and Vivanco, 2009) as well as other various ions, enzymes, water, mucilage, and free oxygen known as root exudates (Afridi et al., 2023). These compounds significantly enhance microbial chemotactic responses, acting as signaling molecules that shape the rhizosphere microbiome (Luo et al., 2020; Koprivova and Kopriva, 2022). They not only initiate microbial colonization of roots but also provide accessible nutrients and energy essential for microbial growth (Zhu et al., 2016).

Interestingly, secondary metabolites such as benzoxazinoids produced by maize roots exhibit selective inhibitory effects on certain bacteria like *Actinobacteria* and *Proteobacteria*, even while some of these compounds are induced to increase plant growth and development (Pascale et al., 2020; Nadarajah, 2018). Activation of mechanisms related to biofilm formation, motility, detoxification, and polysaccharide breakdown triggered microbial recruitment at the root surface. This recruitment catalyzes the growth and niche formation within the microbial community, often through cross-feeding, encouraging the arrival of new bacterial and forming diverse niche groups (Santoyo et al., 2021). Ultimately, plant exudates concentrate efforts on enhancing biofilm production around the roots, reinforcing the intricate web of interactions within the rhizosphere. After microbial communities establish around the root, the release of root exudates into the soil rhizosphere becomes crucial for the belowground interactions between plants and their microbiomes (Mendes et al., 2013). These exudates nourish and shape microbial communities by influencing soil carbon dynamics (root exudates adhere to soil minerals, thereby regulating carbon formation and loss) (Lei et al., 2023). It exudates play an important role in plant-soil communication, functioning as signaling molecules, attractants, stimulants, inhibitors, and repellents, which facilitates plant-microbe interactions (Lamichhane et al., 2023). The specific composition of these exudates directs the assembly of the microbial communities within the rhizosphere (Bulgarelli et al., 2013).

**Fig. 2:**
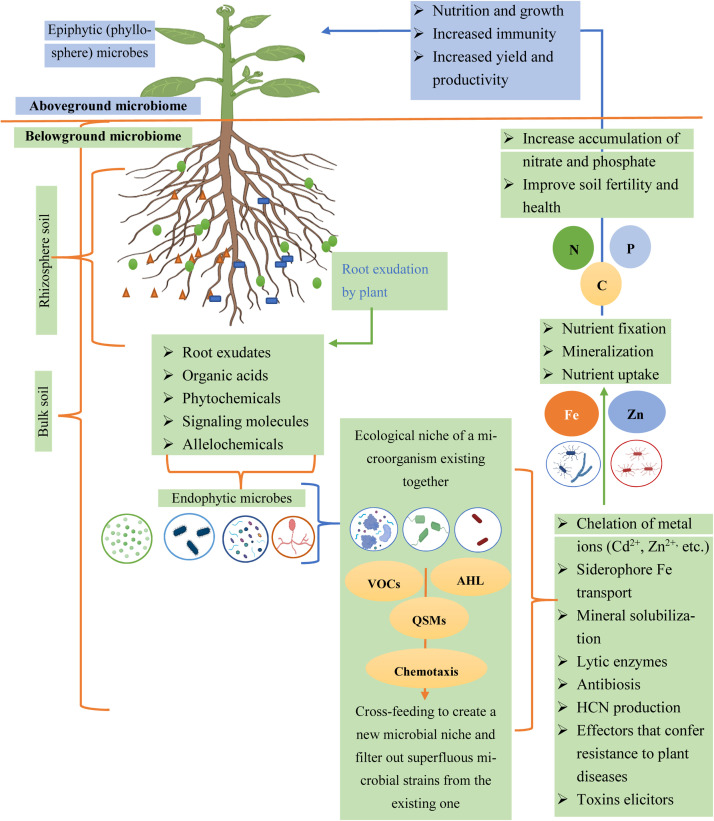
Overview mechanism of plant-microbe interactions: Microbial populations are engaged in creating niches by the root exudates and compounds secreted by plant roots. Allelochemicals, root exudates, and volatile organic molecules may cause the plants to connect with other microbes, creating both advantageous and competitive interactions. While certain metabolites let diverse microbial populations to coexist in the same habitat, others filter away superfluous microbial strains. Through a variety of processes, including the chelation of metal ions (Cd^2+^, Zn^2+^, etc.) to make nutrients available, the transport of siderophores, the production of phytohormones like indole acetic acid, secreted effectors, and antibiotics to shield plants from infections, beneficial bacteria enable the promotion of plant growth. (VOCs-volatile organic compounds; QSM-quorum-sensing molecules; AHL- Acyl homoserine lactones).

The root exudates enhance plant growth and health by attracting beneficial microbes and repelling harmful pathogens (Afridi et al., 2023; Deguine et al., 2023). They also contribute to soil structure and hydration by promoting soil aggregation and retaining moisture (Narula et al., 2012), in addition to aiding in the mobilization of vital nutrients like phosphorus (Hu et al., 2023). The nature of these exudates can be influenced by the plant's genotype, as different species produce distinctive exudates due to their genetic makeup (Hu et al., 2018; Ma et al., 2022). Environmental factors such as soil pH, temperature, nutrient availability, and water retention capacity also impact exudate composition; for instance, plants in phosphorus-deficient soils may increase the release of organic acids and phosphatases to mobilize phosphorus (Gargallo-Garriga et al., 2018). The stage of plant growth significantly affects the quantity and type of exudates produced (Chaparro et al., 2013). Lastly, interactions with other biotic factors can alter the composition of root exudates (Sharma et al., 2023). In essence, these root secretions play a multifaceted role in shaping the belowground ecosystem, influencing everything from microbial communities to soil health and plant growth, all modulated by a variety of intrinsic and extrinsic factors (Fig. 2) (Vishwakarma et al., 2020). Thus, with the influence of root exudates plant demonstrates a diverse arrangement of interactions with the soil-dwelling microbes, such as:

### 3.1 Interactions between plants and microbe

Plant roots release phytochemicals and root exudates, which help microbial communities occupy emerging niches. Selective niches for microbial growth are formed as a result of the relationship between plant roots and microbiome. Certain metabolites remove unneeded microbial strains from environments, prevented from growing in the rhizospheric niche by phytochemicals and root exudates while other metabolites let diverse microbial populations to coexist in the same niche and potentially release chemicals that support the development of other microbes. According to Jacoby and Kopriva (2019), a population that can proliferate through the use of substances released by roots carves out a niche for itself and uses a cross-feeding strategy to attract additional bacteria, creating a new habitat for the remaining microorganisms. For the particular plant species and chemicals being released, a niche selection technique is used. For instance, a number of secondary metabolites with defense characteristics, such as benzoxazinoids released from maize roots, alter the composition of the root microbiome and have the greatest effect on the Actinobacteria and Proteobacteria groups (Hu et al., 2018). It was shown that the combination of substrate selectivity and root exudate composition altered the bacterial population assemblage in the rhizosphere. Fitzpatrick et al. (2018) identified many *Pseudo Xanthomonas* rhizobacterial species that exhibit varying occurrence patterns among thirty angiosperm species, variations in physicochemical conditions and the spatiotemporal organization of the rhizosphere influence the vast diversity and niche specifications of the rhizosphere microbiota (Vetterlein et al., 2020). Numerous endophytic microorganisms also colonize roots internally, and this process is influenced by a number of factors, including the distribution of plant resources and the endophytes' capacity to colonize plants. Because of its usefulness in agriculture, *Piriformospora indica* is one of the major and symbiotic root endophytes. Phosphorus (P) intake is enhanced by the endophyte *P. indica*, which also shields the crop against a range of stressors (Lahrmann et al., 2013). Chemotaxis seeking compounds secreted by roots has been demonstrated by several endophytic fungus. Thus, when non-pathogenic *Fusarium oxysporum* was tested for activity against the root knot nematode (*Meloidogyne incognita*) in tomato plants, it was discovered that the exudates from the plants favorably selected the microbes around the *F. oxysporum* while also facilitating its colonization (Sikora and Dababat, 2007). The bacterial pathogen has been demonstrated in a recent study to adhere to chemotaxis for hydroxylated aromatic acids, which are secondary metabolites secreted by tomato roots (Hasegawa et al., 2019).

### 3.2 Interactions between plant root–root

Allelochemicals, root exudates, and volatile organic molecules can link nearby plants to produce both helpful and competitive relationships. There is more to the root-root relationship than just controlling nutrient uptake. Allelochemicals and root exudates are two examples of the signaling molecules secreted by neighboring plants that facilitate communication between their roots (Mommer et al., 2016a).

Allelopathy is one of them; it is the regular mechanism via which plants communicate by releasing phytotoxins like catechin and provides plants with an edge for competing for limited resources and may be efficiently employed to suppress weeds in the field (Farooq et al., 2013; Andrew et al., 2015), and boost the yearly yield of the soil by developing suitable crop rotation and intercropping systems in comparison to monoculture systems (Cheng and Cheng, 2015). By preventing neighboring plant species from growing, catechin can mediate both intraspecific and interspecific interaction. This reduces competition and increases nutrient availability (Mommer et al., 2016b). In an experiment conducted over two years, intercropping sorghum (*Sorghum bicolour* L.), sesame (*Sesamum indicum* L.), and soybean (*Glycine* max L.) in a cotton field (*Gossypium hirsutum* L.) resulted in higher net benefits and a significant inhibition of weed purple nutsedge (*Cyperus rotundus* L.) compared to the cotton alone (Iqbal et al., 2007). Through allelopathy, in which produced phytotoxins are able to decrease the development or survival of neighbors and, thus, lessen competition for resources, these interactions also affect the root growth of nearby plants. According to Wang et al. (2015), relay intercropping aubergine and garlic is a useful agricultural technique that sustains greater aubergine development and increased output. Increased system-level competitiveness and allelopathic interactions can lessen the severity of weeds, pests, and diseases, thereby increasing yield.

As allelochemicals, volatile organic compounds also control mycorrhiza networks' rhizosphere signaling availability (Mommer et al., 2016b; Nadarajah, 2017). Due to their inherent resistance to their own phytotoxins, allelopathic plants exhibit varying degrees of specificity when it comes to their effects on other plant species.

Numerous interactions are impacted by the belowground processes in the plant-soil system, including nutrient cycling, plant interspecific competition, pest and disease attenuation, soil community composition and structure, and soil structural dynamics (Ehrmann and Ritz, 2014). Hauggaard-Nielsen and Jensen (2005) state that later in the growing season, early interspecific below-ground rivalry may change to facilitation, which further increases root development and activity. This might be the reason why multi-cropping systems often encourage root growth as compare to monoculture (Yong et al. 2012). Growing mixtures of different crops together can lead to higher productivity and pest tolerance. Plant-root interactions in polyculture systems increase resource sharing through common mycorrhizal fungal networks, recycling nutrients through leaf senescence, and root turnover (Brooker et al., 2014); niche complementarity considering plants with different shoot and root architectures can better exploit light and soil resources (Postma and Lynch, 2012); provide protection against mineral toxicities in saline, sodic, or metalliferous soils (Inal and Gunes, 2007; White and Greenwood, 2013); decrease pest and disease losses as the increased diversity in polyculture can decrease pest and disease losses (Bracken, 2018); increase resource use efficiency in time and space (Ehrmann and Ritz, 2014); and lower crop failure risk because polyculture mimics natural ecosystems and improves ecological resilience (Iverson et al., 2014). Allelopathy should thus be experimented with appropriately to increase agricultural yield and safeguard the environment by controlling weeds, insect pests, and crop diseases in an ecologically responsible manner.

Plant genotypes and species exhibit considerable exudate specificity, which might affect nearby plants. Numerous mechanisms, such as affecting nutrient availability (Canarini et al., 2019) and moderating nutrient competition, have been linked to these root exudates. Beyond this, many investigations were conducted to demonstrate disparate pieces of data on the interactions between plant roots and distinct niches. For example, vertically distributed roots are not associated with integral niches, but rather with competitive interactions amongst plants, as demonstrated by Semchenko et al. (2018). According to their findings, there is intense rivalry among plant species, which causes some of them to extend their roots and inhibit nearby species. In contrast, species with fewer branching and deeper root systems are better equipped to endure this competition. The rhizobiome regulates the exudates around the roots, impacting their amount, composition, and potential for interaction between the microbiome and plant. It would seem from the aforementioned fact that the root-root interaction is competitive rather than not niche (Semchenko et al., 2018). Species with more widely distributed horizontal root systems than those with deeper, more vertical root systems exhibit competition between adjacent plants. Furthermore, Weidlich et al. (2018) demonstrated facilitative interactions between the subsurface roots of non-legume and legume species utilizing genetically modified plants. These interactions are restricted to interactions between genotypes as well as between different species. Moving from interactions between species to interactions between genotypes, Montazeaud et al. (2018) conducted experiments on a number of species and measured the productivity of pairs of rice plants (*Oryza sativa*). They found that mixture productivity in crops increased with an increase in between-genotype distance, which was explained by resource-use complementarity. The significance of direct competition over the niche complementarity theory was further supported by these findings.

### 3.3 Interactions between plant root and microbe

Plant species identification has a significant impact on the range diversity of organisms that live in soil, especially those that are around the plant (Kowalchuk et al., 2002). The association between the root microbiome and the plant is one of the most extensively researched relationships, along with those between rhizobacteria and legumes, actinobacteria and roots, mycorrhizal roots, and other root-microbe interactions. It is possible to discuss the root-microbe interactions as both symbiotic and parasitic relationships. In order to have a symbiotic relationship with plants, microorganisms release a variety of beneficial chemicals into the rhizosphere for plant absorption. These chemicals help to control the transcriptome of plants. A number of cytokines, auxins, and gibberellins are produced by the microbial community that lives close to plant roots in addition to the hormones that plants produce (Fahad et al., 2015). Beneficial bacteria facilitate plant growth through a variety of processes, including chelating and transporting nutrients to plants (*e.g*., the siderophore–Fe transporter, which carries iron that is usable) and generating phytohormones (*e.g*., indole acetic acid), secreted effectors, and antibiotics to shield plants from disease. N-acyl homoserine lactone (AHL); molecules that sense quorum; volatile organic substances; iron, zinc, and copper. Root exudates specific to a given plant show the particular selection of rhizospheric microbial communities. For example, the fumaric acid secreted by banana roots attracted *Bacillus subtilis* towards roots, resulting in the formation of biofilms, and the cucumber plant secreted citric acids from its roots, which in turn influenced the attraction of *Bacillus amyloliquefaciens* (Zhang et al., 2014). According to Hassan and Mathesius (2012), these substances play a significant role in imitating quorum sensing in bacteria, which has an effect on bacterial metabolism. In addition to these, a number of other substances, such as tryptophan, which is involved in the biosynthesis of indole acetic acid (IAA), aid in the synthesis of phytohormones needed by bacteria for PGPR activities (Haichar et al., 2014). Certain substances, such as flavonoids, which are 2-phenyl-1,4-benzopyrone derivatives, have been shown to be able to induce the formation of nodules in roots. When flavonoids interact with the constitutively expressed internal proteins of rhizobial regulatory nodD genes to form a transcriptional activator of other nod genes whose protein products are responsible for the synthesis of reciprocal signal molecules to the host plant root—the chitolipooligosaccharide (LCOs) Nod factors (Cooper, 2004). This interaction constitutes the first of many elements that influence host specificity in legume-rhizobia symbioses and is a critical step in the infection process. LCOs Nod factors are essential signals for rhizobial entry into legume roots (Relic´et al., 1994), and the success or otherwise of the infection process is in large part determined by their structural features. The perception of Nod factors by the plant is mediated with the specific receptors. They are represented by transmembrane serine/threonine receptor-like kinases with extracellular domains containing three LysM motifs (Madsen et al., 2003; Radutoiu et al., 2003). In *Lotus japonicus,* the receptors are encoded by the genes Nod factor receptor kinase 1 and 5 (LjNfr1 and LjNfr5). In *Medicago truncatula* and *Pisum sativum* orthologous pairs of the genes Nod factor perception (MtNfp) and PsSym10 (Arrighi, et al., 2006) and LysM domain-containing receptor-like kinase (MtLYK3) (Limpens et al., 2003) and PsSym37 (Zhukov et al., 2008) were observed. The first morphological changes observed on legume plants of Nod factors are deformations of root hairs (Lerouge et al., 1990) followed by curling of the root hairs (Ehrhardt et al.,1992; Felle et al., 1995). Root hair curling leads to entrapment of a single cell of rhizobia. Bacterium inside root curling actively divides, which leads to the formation of a microcolonies, which in turn develops within the infection chamber (pocket), gradually increasing in size, and which is accompanied by a reorganization of the infection chamber. Secondly, rapid increases then oscillations in intracellular free calcium in root hairs, often referred to as calcium spiking (Ehrhardt et al., 1996; Wais et al., 2002; Walker et al., 2000). They are accompanied by an active reorganization of the actin and tubulin cytoskeleton (Pandey et al., 2019; Poudel et al., 2019) and the movement of the nucleus in the root hair cell. The further stage of infection is the preinfection thread formation in deformed root hairs (van Brussel et al., 1992); and formation of an infection thread (IT), a special tubular structure that ensures the penetration of rhizobia into the root (Brown et al., 2020; Sivakumar et al., 2020). In parallel with the induction of the infection process in the root, localized cortical cell divisions are activated, resulting in the formation of nodule primordium (Agler et al., 2016). When the IT reaches the primordium cells, specialized outgrowths of the IT, devoid of cell wall and surrounded by only a plasma membrane, called infection droplets are formed (Brown et al., 2020). From these outgrowths, bacteria are released into the plant cell cytoplasm. Bacteria are separated from the cytoplasm by a peribacteroid (symbiosome) membrane. As a result of differentiation of rhizobia, a specialized nitrogen fixation form, called bacteroid, is formed. A bacteroid surrounded by a peribacteroid membrane is known as a symbiosome (Frank et al., 2017).

The development of ITs is not limited to infection of the nodule primordia. In temperate legumes, such as *P. sativum, Medicago sativa, Vicia faba*, etc., nodule meristem functions for a long time, and such nodules are called indeterminate nodules. As a result of the meristematic activity, new cells constitutively leave meristem and can be infected. These cells form an infection zone into which ITs penetrate and grow, reaching meristematic cells. However, in the nodule without bacterial release in some cells, infection droplets still form (Santi et al., 2013) and bacterial release is observed (Nouioui et al., 2019). The development of abnormal ITs leads to the activation of defence reactions manifested in their suberinization (Adeleke et al., 2019a). In L. *japonicus*, the mutant Ljalb1 (aberrant localization of bacteria inside nodule) formed two types of nodules: in small white nodules, hypertrophied ITs were formed, lacking bacterial release (determinate nodules) and in pale pink nodules infected cells were formed (Odoh et al., 2019). When there is an adequate supply of nitrogen, this process is controlled by feedback inhibition, which stores energy and prevents N_2_-fixation (Pankievicz et al., 2019). The signaling compounds produced by the rhizobacteria affect their host.

Research conducted on *Medusa truncatula* revealed that the nodule's protein composition changed when leghemoglobin and enolase isoforms formed ethylene-responsive proteins were induced by *R. leguminosarum* in different legumes (Trouvelot et al., 2014). Plant defense response is significantly regulated by ethylene. Hyper nodulation was seen in the roots of the *M. truncatula* (skl) ethylene-insensitive mutant, most likely as a result of weakened immunity. The ethylene pathway of the skl mutant was said to be malfunctioning. Thus, the propose that ethylene is the cause of the host's symbioses and nodulation. The connection between rhizobacteria and legumes is also influenced by nutrient exchange. In plant symbiosomes, rhizobacteria are found as bacteroid. The bacteroid and peribacteroid membranes composition regulates all nutrient exchange. Actinomycetes can coexist harmoniously with their host plants in symbiotic partnerships. There have been reports of several symbiotic connections between this group of bacteria and Angiosperms, including the genera *Datisca, Alnus*, and *Casuarina* (Sellstedt and Richau, 2013). Studying the proteomes of *Alnus* sp. and *Frankia alni* revealed secreted proteins, most of which were hydrolytic enzymes thought to be important for the development of this symbiotic association (Santi et al., 2013). Mycorrhizal fungi are another class of organisms that live in symbiotic relationships with plants. These fungi enter the root systems and form an arbuscular in the extracellular hyphal structures (ectomycorrhiza or EM) or cortical cells. Among these fungal interactions, the AMF are the most prevalent (Santi et al., 2013; Nouioui et al., 2019). Similar to rhizobacteria, AMF is kept apart from the plant by a membrane that does not impede the fungus and host's ability to exchange nutrients. In return for carbon and fats, AMF gives the plant phosphorus (Nadarajah and Kumar, 2019). To prevent the symbiont from losing too much nutrition from the host, the carbon supply is feedback-regulated (Begum et al., 2019). A proteome study of the *M. truncatula* root colonized by *Glomus mosseae* revealed redox, stress, respiration, and cell wall modifications—all of which are required for *Glomus mosseae* to colonize the host root system. When *G. intraradices* was injected into *M. truncatula* proteins, both wild type and mutant [dmi3] proteins showed distinct protein expression patterns (Amiour et al., 2006). Using proteome and transcriptome analysis, proteins including ATPases, lipoxygenases, and thioredoxins were found. Subsequent research on these proteins revealed the significance of metabolism (amino acids, fatty acids, and carotenoids) and transporters (nutrients and water) in AMF-infected roots (Lai et al., 2018).

Root exudates have a significant role in phytoremediation because they help host plants actively adapt to and survive in the presence of metal stressors through a variety of processes, such as allelopathic functions that affect the growth of rhizosphere microbes and other plants or detoxification processes that include the adsorption, chelation, transformation, and inactivation of metals. And more, root exudates—especially organic acids—have the potential to bind metalions, which affects the metals' mobility, solubility, and bioavailability in soil (Chiang et al., 2011; Luo et al., 2014). According to Hartmann et al. (2009), plants have the potential to choose their own root microflora from their environment. As a result, each type of plant has a unique group of related bacteria. This mechanism is most likely directly related to the amount and makeup of root exudates as well as the characteristics of the soil in the rhizosphere. Plant-microbe interactions are very dynamic by nature, as a result of co-evolutionary forces (Chaparro et al., 2014). By releasing chemicals or signals (signaling molecules and their perception, quorum sensing) into the rhizosphere, plants can effectively communicate with nearby soil microorganisms. Meanwhile, their associated microbes can form an efficient associative symbiosis with plants by inducing host functional signals (e.g.*,* microbial chemotaxis and colonization) (Doornbos et al., 2012; Drogue et al., 2012; Bulgarelli et al., 2013).

## 4 Plant-microbe interaction is bidirectional

Plants and microbes interact in both directions which includes reciprocal relationships that can either be beneficial or detrimental to both. Microbes that may settle in apoplastic spaces, plant surface areas, or regions close to the plant surface, such as the rhizosoil, find refuge in plants. Between the host and the microbe, there is mutualism—an interaction in which the plant and the microbe benefit from one another without causing harm—as well as commensalism (Sharma et al., 2021). Apart from providing a protected environment, numerous plants emit substances that draw in and nourish the related microorganisms (Bulgarelli et al., 2013). Consequently, these mutualistic microorganisms could release substances that promote plant development and supply the plant with nutrients (Berendsen et al., 2012). Additionally, they might strengthen the plant's resistance to biotic or abiotic stressors or act as a barrier against infections (Pineda et al., 2010). Plant-microbe interactions are extremely dynamic and complicated, encompassing a wide range of mutually beneficial interactions, such as:

### 4.1 Sheltered habitat

The microbiome of plants is a dynamic consortium made up of all the microbial genomes that colonize various tissues in the rhizosphere, phyllosphere, and endosphere. These microorganisms include bacteria, fungus, viruses, and nematodes. Throughout their structures, plants offer a variety of sheltered habitats for microorganisms. The spaces between plant cells, or apoplastic spaces, are regions that microbes can occupy within a plant. They may also live on the surface of the plant in places like the leaves, stems, and roots. It is well recognized that microbes live in the plant reproductive system, or flowers, and that they are essential to the preservation of plant species. Different bacteria have their own habitat provided by floral structures such as the calyx, corolla, ovaries, stamens, stigma, and style (Junker et al., 2011; Ottesen et al., 2013). Microbes live within plant fruits (endophytes) as well as outside (epiphytes). *Brachybacterium, Chryseomonas, Microvirga, Microbacterium, Microbacteriaceae, Paracoccus, Rhizobium*, and *Sphingomonas* are among the microbial species that live in flowers and fruits (Ottesen et al., 2013). In addition, one of the best places for microbial colonization is the rhizosphere, or the soil around plant roots. Microbes can find a stable environment and nutrients released by plant roots in these places (Bulgarelli et al., 2013). Numerous exudates secreted by plant roots in the rhizosphere serve as microbial attractants and ultimately enhance the physicochemical characteristics of the surrounding soil. However, these exudates preserve the microbial communities' structure and function close to plant roots (Khan et al., 2021; Pandey et al., 2017). Microbes and plants coexist in symbiotic relationships.

### 4.2 Attraction and feeding

A variety of substances released by plants, including sugars, amino acids, and organic acids, operate as both attractants and nutrients for microorganisms that are connected with them. These substances frequently leak into the rhizosphere through the roots. According to Berendsen et al. (2012), this process, called rhizodeposition, is essential for creating a symbiotic community of helpful microorganisms surrounding the plant. A significant part of the carbon cycle is played by rhizodeposition. About 25 % of the carbon that the plant obtains during photosynthesis will be released through the soil's roots; this carbon is what many microbes rely on. Photosynthetic energy is a major source of energy for a plant's microbiome. Plants employ this process to transform carbon dioxide, water, and sunlight into food and oxygen in the form of sugars, which are partially carbon-containing molecules. Through their roots, plants release these sugars as well as other substances. In the microbiome, fungi and other bacteria aid in the breakdown of organic materials into basic chemical compounds that are useful to plants and other microbiome creatures. The researchers hypothesize that plants occasionally use chemicals to interact with their microbiomes.

### 4.3 Mutualistic connections

The ecological relationship between two coexisting species is known as symbiosis. Mutualistic refers to a symbiotic connection in which both parties to the association gain from it. Certain microbes develop beneficial mutualistic connections with plants in exchange for the nourishment and shelter the plants supply. Utilizing interactions between plants and microbes, plant growth has been enhanced for the production of food, fiber, biofuels, and essential metabolites. Indirect nutrient delivery to the plant (biofertilizer) or enhanced availability of nutrients like iron or phosphate are two advantages of the mutualistic relationship. Legume symbiosis with nitrogen-fixing rhizobia and the symbiosis of plants with arbuscular mycorrhizal (AM) fungi, are one of the most common plant-microbe interaction. Many other kinds of microbes, such as cyanobacteria, algae, plants, and mammals, have mutualistic relationships with fungi. Almost all vascular plant species have mycorrhizal partners; this number approaches 90 %. The fungal mycelia in a mycorrhizal relationship carry water and minerals from the soil into the plant through their vast network of hyphae and enormous surface area in contact with the soil. In return, the fungus uses the plant's photosynthetic byproducts as fuel for its metabolism. Although phosphorus, nitrogen, and other mineral nutrients seem to be the main functions of AM symbiosis, the plant also gains disease resistance from this symbiotic relationship. As an illustration, mycorrhizal fungi and plant roots develop symbiotic interactions that improve the plant's capacity to take in water and nutrients from the soil (Bulgarelli et al., 2013). Undoubtedly one of the most well-known mutualistic plant-bacteria interactions is the symbiosis between legumes and *Rhizobium*. Through a process known as symbiotic nitrogen fixation, rhizobia colonize the plant root and supply the plant with nitrogen. A signal exchange between the host plant and its microsymbiont initiates the legume-rhizobial symbiosis (Oldroyd, 2013). Rhizobia, which are soil bacteria that fix nitrogen, may coexist harmoniously alongside legumes. This symbiotic relationship causes the bacteria to create nodules on the plant root, where they can transform atmospheric nitrogen into ammonia, which the plant may use for growth and development. According to Pineda et al. (2010), rhizobia bacteria transform atmospheric nitrogen into a form that plants can exploit. Auxins and cytokinins, two compounds that promote growth and are produced by plant growth-promoting rhizobacteria (PGPR) (Lugtenberg and Kamilova, 2009).

### 4.4 Stress resistance

Plants have established multi-level stress resistance mechanisms during the course of evolution that can be used coordinately against abiotic factors (salt, drought, heavy metals, nitrogen shortage, and extreme heat or cold). Abiotic stress has a major impact on plant development and is a major contributor to considerable losses in agricultural goods (Gonzalez-Guzman et al., 2022; Ghadirnezhad Shiade et al., 2023) which reported for more than 50 % (Rejeb et al. 2014). The use of high salinity water for agriculture and the fast expansion of different sectors are two other significant issues that have an impact on crop yield. According to Slama et al. (2015), there is also a connection between excessive salinity and drought, which is a significant problem that can be exacerbated by very variable temperatures. (Wahid et al., 2007; Huang; et al., 2012; Djanaguiraman et al., 2018) reported that, high temperatures can stress proteins severely, cause protein synthesis, enzyme inactivation, membrane damage, induce root growth, oxidative damage, and reduce photosynthetic rate. Plants may adapt and become resistant to a variety of abiotic stimuli, such as salt, heavy metals, drought, and nutrient deprivation, thanks in large part to phytohormones such as auxins, gibberellins, and cytokinins that alter the shape of the roots (Michael, 2021). PGPM are considered as bio-ameliorators which aids plants in alleviating drought and salt stress. According to a study by Hidri et al. (2022) inoculation of *Glutamicibacter* sp. significantly reduced malondialdehyde (MDA) concentration, while increasing the activity of antioxidants enzymes (superoxide dismutase; catalase; ascorbate peroxidase; and glutathione reductase) by up to 100 % to help scavenge reactive oxygen species (ROS) that can harm plant cells. The PGPM helps in secretion of auxins, including indole acetic acid (IAA), increases cell elongation, which boosts root growth and encourages the production of lateral roots to help plants absorb more water and nutrients (Sing et al., 2024). By inducing systemic tolerance (IST), which involves the synthesis of antioxidants (superoxide dismutases, peroxidase, ascorbate peroxidase, catalase, and glutathione reductase), and the breakdown of the ethylene precursor 1-aminocyclopropane-1-carboxylate (ACC) by bacterial ACC deaminase, PGPM helps to lessen the harmful effects of abiotic stress (Farooq et al., 2009; Porcel et al., 2014) and heavy metals and acidity (Naamala and Smith, 2020). The increase in the production of low-molecular-weight osmolytes, such as glycine betaine, proline and other amino acids, organic acids, nitrogen fixation, mineral phosphate solubilization, and the production of important enzymes like ACC-deaminase, chitinase, and glucanase, microbes can in fact support plant growth and development under conditions of abiotic stress (Ahmad et al., 2011; Gupta et al., 2013). By increasing water intake and stress-related gene expression, endophytic bacteria can increase a plant's resistance to drought (Compant et al., 2022). PGPR synthesizes exopolysaccharide (ESP), extracellular polysaccharides (EPS), various osmolytes, alters root morphology, as well as maintaining osmotic balance and ion homeostasis which shield plant cells to help prevent excessive water loss (Gupta et al., 2022). In addition, the signal transduction pathway and reactive clearance of oxygen are crucial mechanisms for coping with drought stress via calcium and phytohormones such as abscisic acid, salicylic acid, jasmonic acid, auxin, gibberellin, ethylene, brassinosteroids and peptide (Iqbal et al., 2022). Producing antibiotics which shield plants from soil infections and osmolytes assist shape the water content in plant and bacterial cells (Gamalero and Glick, 2022).

### 4.5 Pathogen defense

Microbes frequently contribute to the complex immune systems that plants have evolved to fend off infections. Several pathogenic microbes that threaten plant growth and development are frequently encountered by plants at various phases of their life cycle. Through signaling pathways that are sensitive to various biotic and environmental cues, plants adapt to these stressful situations by changing at the transcriptome and metabolomics levels. In order to maintain growth and productivity, plants use a variety of adaptive response strategies (Compant et al., 2022), including increased production of primary and secondary metabolites, reactive oxygen species (ROS) signaling (Sahu et al., 2022), phytohormone production, modifications to plant hydraulic status, and osmotic regulation (Yadav et al., 2021). Pathogens are impeded in their ability to establish colonies in plants by competition from other organisms for resources and space (Berendsen et al., 2012). After being exposed to pathogens, plants frequently establish immunological memory, which results in induced resistance. Induced resistance is a state of enhanced defensive mechanism that is developed by a plant when it is stimulated appropriately. Induced systemic resistance (ISR) and systemic acquired resistance (SAR) are two types of induced resistance in which the defense mechanism of the plant is preconditioned by previous infection or treatment, resulting in resistance against a subsequent challenge by a pathogen or parasite (Choudhary et al., 2007; Kour et al., 2023). ISR is of great interest from an agronomical perspective due to its efficacy against a wide range of phytopathogens (Prsic and Ongena, 2020). ISR is frequently linked to the plant hormones ethylene (ET) and jasmonic acid (JA) (Kaur et al., 2022) which strengthens the plant's defenses against diseases by boosting its chemical or physical barriers. Salicylic acid (SA) and pipecolic acid (Pip), two plant hormones, are frequently linked to SAR (Mishra et al., 2024; Llorens et al., 2020) and prepares the plant's healthy leaves for pathogen defense. These plant hormones signaling molecules as well as plant messenger signaling molecules are important in triggering plant defense mechanisms such as pathogen-associated molecular pattern-triggered immunity (PTI) and effector-triggered immunity (ETI) (Ding et al., 2022) to stop the proliferation of dangerous microorganisms (Nishad et al., 2020). PGPM such as PGPR and PGPF provides shelter for friendly beneficial microbes within the plant body, increasing the plant's resilience to pathogenic microbes. These beneficial microbes protect from pathogenic diseases by synthesize toxins against invading harmful microbes. This type of plant–microbe interaction is often called a symbiotic association. Mutualistic plant-microbe interactions include associations with mycorrhiza, rhizobium, and endophytic bacteria, which trigger a plant immunological response. Mycorrhiza-induced resistance is a superior defense mechanism that plants acquire in response to arbuscular mycorrhizal fungi (AMF) (Cameron et al., 2013). By inducing systemic resistance, AMF inhibits plant diseases and pests (Jung et al., 2012; Song et al., 2015). According to Radzki et al. (2013), PGPR can control a variety of plant diseases by denying pathogens iron, which inhibits the development of illness and produces extracellular siderophores. The three most effective strategies for a possible biocontrol agent are bacteriocins, siderophores, and antibiotics (Simons et al., 2020). The synthesis of plant-beneficial metabolites like siderophores has been studied in a number of studies as a possible method for managing plant diseases (Subramanian and Smith, 2015). Rhizobacteria such as *Azospirillum, Azotobacter, Bacillus, Rhizobium, Serratia, Stenotrophomonas, Streptomyces Acinetobac-ter, Agrobacterium, Alcaligenes, Arthrobacter, Bradyrhizobium, Frankia, Pantoea, Pseudomonas,* and *Thiobacillus* are important for biological control of plant pathogenic microorganisms (El-Saadony et al., 2022). Plants have a different kind of induced immune response through mutualistic interaction with friendly microbes. This reaction is brought about by giving benevolent microorganisms a home within the plant's body, which helps the plant develop resistance against pathogenic germs. Toxins produced by the helpful bacteria shield the plant against dangerous pathogens and prevent them from invading. In natural and agricultural environments, interactions between microbes and plants are essential for plant growth, resilience, and overall health.

## 5 Plants depend on beneficial microbes

Pathogens can severely affect agricultural production, hence, to tackle these challenges, strategic measures should be taken in the agricultural management system. One of the strategies is the application of PGPMs in agriculture. The beneficial microorganisms such as PGPR and PGPF, which interact with plants to promote their development and well-being, are the subject of several research on plant-microbe interactions. PGPMs are a common type of microbe found in nature. These PGPMs are a particular class of microorganisms connected to the roots that support plant growth and shield plants from diseases and abiotic stress. PGPMs have been shown in several studies to improve the metabolic response in plants in a species-specific way (Dhawi et al., 2017; Pratush and Kumar, 2018). Plant-associated microbial populations, such as PGPM, which support plant growth and development under a variety of abiotic and biotic conditions, are one of the beneficial options (Tank and Saraf, 2010). As PGPM activates the cellular component and accumulates secondary metabolites, it has been regarded as an economical and ecologically benign way to manage stress (Fahad et al., 2015). It is well recognized that PGPM is crucial for plant development and metabolism, helping to sustain plant growth and development under adverse circumstances.

### 5.1 PGPM as microbial inoculants/biofertilizers

**Fig. 3:**
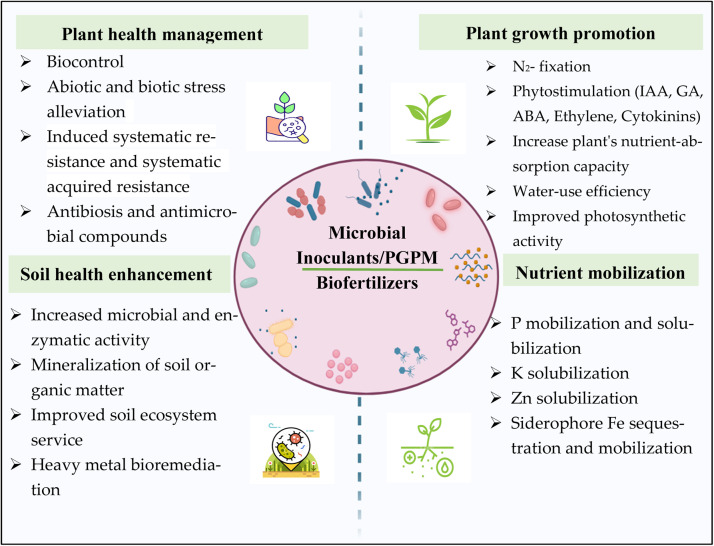
Mechanism of PGPM/microbial inoculants as biofertilizers: As microbial inoculants or biofertilizers, PGPM that are extracted from the soil or plant rhizosphere can be employed to regulate plant health, nutrient mobilization, and enhance he plant productivity and soil health.

A sustainable way to increase crop yields is to utilize beneficial microorganisms, also known as PGPMs, as microbial inoculants, or biofertilizers (Table 1). PGPMs, which includes cyanobacteria, endo- or ectomycorrhizal fungi, rhizobacteria, soil or endophytic bacteria, and many more, may colonize soil and plants in large amounts (10^5^–10^7^ CFU per gram of fresh root) and benefit plants in a number of ways (Mishra et al., 2017). In addition to acting as biocontrol agents against plant diseases and pests, they can enhance nutrient absorption, plant development, and plant tolerance to abiotic and biotic stress (Gangwar et al., 2017; Valenzuela-Aragon et al., 2018). To ensure PGPM application as agricultural inputs, it is imperative to make it available to farmers. A number of studies have shown how PGPF (Hossain et al., 2017) and PGPR (Malgioglio et al., 2022) that are isolated from the soil or plant rhizosphere can be used as biostimulants, biofertilizers, and inducers of resistance against a variety of biotic and abiotic stresses efficiently and enhances the plant productivity and soil health (Nadarajah and Rahman, 2021) (Fig. 3). By bio-fixing atmospheric nitrogen and solubilizing soil minerals like phosphorus and potassium, PGPM function as a biofertilizer, boosting the availability of nutrients (Bhat et al., 2019). The majority of microbe-based inoculants come from the subgroups of fungus (particularly *Trichordema*) and bacteria (such *Bacillus* and *Rhizobia*) (Hartmann et al., 2014), while other archea groups have also been shown to augment plant development (Berg and Koskella, 2018; Bashan et al., 2013). Microbial inoculants typically consist of a mixture of live microorganisms and/or their components, such as parts or compounds, and a nonliving carrier, which can take the form of a liquid or solid (Alori and Babalola, 2017, 2018). Microbial cells can be dormant or active; if the latter, they must be reactivated either before to or upon inoculation (Babalola and Glick, 2012a). Moreover, they might be pure cultures (single strains) or microbial consortiums, which are collections of several microbial strains (Alori and Babalola, 2018). Potential PGPMs strains should have attributes like the capacity to grow plants through a variety of mechanisms, the capacity to establish populations and compete favorably in the rhizosphere, the ability to persist throughout the seasons in the rhizosphere, and the capacity to be grown in artificial environments (Babalola and Glick, 2012; Khandelwal et al., 2002). The inoculant's mode of application is frequently determined by the formulation type and manner. For example, liquid formulations may be administered in a variety of ways whereas solid formulations are mostly applied via broadcasting over the field or seed dressing (Khandelwal et al., 2002). Water and/or organic solvents (apart from microbiological media) like glycerol and carboxymethyl cellulose are the most common liquid carriers; these substances are used to improve characteristics like stickiness and dispersion capabilities (Bashan et al., 2013). Solid carriers come in a variety of forms, including clay, vermiculite, peat, and charcoal (Babalola and Glick, 2012). When choosing microbial carriers, care should be made to make sure they won't harm the germ or the environment (Alori and Babalola, 2018). When it comes to discovering a source of beneficial bacteria, harsh environments are very intriguing. The microbe's chances of surviving in the field can be increased by formulation, which can protect them from unfavorable environmental conditions, lengthen their shelf life, and meet their nutrient requirements (Bashan et al., 2013; Berninger et al., 2018).

**Table 1:** Microbial formulation developed in relation to different agricultural purposes.

Microbial inoculants used	Trade name	Origin /Producer	Applications/ Uses	Reference
*Bradyrhizobia*	Nodumax	IITA, Kenya	Nitrogen fixation	Tairo and Ndkidemi (2014)
*PGPR consortia*	Vita Soil	Symborg, Spain	Nitrogen fixation and Rhizosphere microbial population enhancer	Sekar et al. (2016)
*Azospirillum* sp.	Ajay Azospirillum	Ajay Biotech, India	Nitrogen fixation	Celador-Lera et al. (2018)
*Azobacterium brasilense*	Azobacterium	JSC Industrial Innovations, Russia	Nitrogen fixation	Celador-Lera et al. (2018)
*P. aurantiaca*	Liquid PSA	Laboratorios BioAgro S.A., Agrentina	Nitrogen fixation and Biocontrol	Celador-Lera et al. (2018)
*Rhizobium* sp.	Nitrasec	Lage y Cia, Uruguay	Nitrogen fixation	Adeleke et al. (2019)
*Mesorhizobium cicero*	Chickpea Nodulator	Becker Underwood, United States of America	Nitrogen fixation	Adeleke et al. (2019)
*Rhizobia*	Biofix	MEA Fertilizer Ltd, Kenya	Nitrogen fixation	Adeleke et al. (2019)
*A. brasilense, Azotobacter vinelandii, B. megaterium*	BactoFil A10	AGRObio, Hungary	Nitrogen fixation and Biocontrol	Mustafa et al. (2019)
*Azotobacter, P. fluorescens*	Biomax	Greenmax AgroTech Life, India	Nitrogen fixation and biocontrol	Odoh et al. (2019)
*B. amyloliquefaciens IT 45, B. japonicum*	Rhizocell GC Nodulator	Lallen and plant care BASF Inc., Canada	Nitrogen fixation and Biocontrol	Odoh et al. (2019)
*Rhizobium, Enterobacter* spp.*, Bacillus* spp.*, Stenotrophomonas, Pseudomonas*	Organico	Amka Products (Pty) Ltd, South Africa	Nitrogen fixation, Phosphorus solubilisation and Biocontrol	Adeleke et al. (2019)
*Azospirillum* sp.	Azospirillum-Biofertilizer	AAU, Assam, India	Nitrogen fixation	Nath et al. (2020a)
*Azotobacter* sp.	Azotobacter-Biofertilizer	AAU, Assam, India	Nitrogen fixation	Nath et al. (2020b)
*Azotobacter chroococcum*	Bio-Bacter	ICAR- NBAIM, India	Nitrogen fixation	Saxena et al. (2020)
*Rhizobial strains*	RhizoNBAIM	ICAR- NBAIM, India/ Agrinnovate India Ltd.	Nitrogen fixation	Saxena et al. (2020)
*Bradyrhizobium japonicum*	HISTICK	BASF SE, Germany	Nitrogen fixation	Mehnaz (2016)
*B. japonicum*	BIODOZ	Novozymes, Denmark	Nitrogen fixation	Berninger et al. (2018)
*B.japonicum*	Cell-Tech	Monsanto (Bayer), Belgium	Nitrogen fixation	Berninger et al. (2018)
*Trichoderma and Bradyrhizobium* *Spp. (Excalibre‐SA) consortium*	Bio Grow	ABM, USA	N fixation and Growth stimulation	Backer et al. (2018)
*Azotobacter chroococum + Paenibacillus tylopili + Bacillus decolorationis*	Bio NPK	ICAR, India/ Prathista Industries Ltd., Secundrabad	Nitrogen fixation, Phosphorus solubilisation, Potassium solubilisation	Saxena et al. (2020)
*Bacillus megaterium*	Bio Phos	Bio Power Lanka, Sri Lanka	Phosphorus solubilisation	Mehnaz (2016)
*Pseudomonas striata, B. Polymyxa and B. megaterium consortium*	P Sol B	Agrilife, India	Phosphorus solubilization	Mehnaz (2016)
*B. polymyxa, B. subtilis*	InomixR	Lab (Labiotech), Spain	Phosphorus solubilisation	Odoh et al. (2019)
*Kluyvera cryocrescens + Paenibacillus tylopili*	Bio Phos+	ICAR- NBAIM, India/ Agrinnovate India Ltd.	Phosphorus solubilisation	Saxena et al. (2020)
AMF consortium (*Glomus mosseae + Glomus intraradices + Glomus* sp.)	AM Fungal Biofertilizer	ICAR- IISR, India	Phosphorus solubilisation	Saxena et al. (2020)
*Bacillus* sp. *BC39 + Bacillus* sp. *RC25 + Pseudomonas* sp. *K30 + Pseudomonas* sp. *K31*	BIOGROW	ICAR- NBAIM, India/ Agrinnovate India Ltd.	Phosphorus solubilization, IAA and siderophore production	Saxena et al. (2020)
*Bacillus decolorationis*	Bio Potash	ICAR- NBAIM, India/ Agrinnovate India Ltd.	Potassium solubilisation	Saxena et al. (2020)
*Pseudomonas gessardii BHU1(PGPR1) + Pseudomonas putida S1(6) (PGPR2) + Pseudomonas* *aeruginosa BM6 (PGPR4)*	NutBoost	ICAR-DGR, India	P, K, and Zn solubilisation	Saxena et al. (2020)
*Acidithiobacillus ferrooxidans*	Fe Sol B	AgriLife	Iron mobilization	Mehnaz (2016)
*Arthrobacter* sp.	ZincFort	ICAR- NBAIM, India	Zinc solubilisation	Saxena et al. (2020)
*Bacillus endophyticus*	Bio Zn	ICAR- NBAIM, India/ Agrinnovate India Ltd.	Zinc solubilisation	Saxena et al. (2020)
*Arthrobacter sulfonivorans*	IronFort	ICAR- NBAIM, India	Growth stimulation	Saxena et al. (2020)
*Kappaphycus alvarezii*	Sagarika	IFFCO, India	Plant growth promotion	Karthikeyan and Shanmugam (2016)
*30 bacterial species*	Inogro	Flozyme Corporation, United States of America	Plant growth promotion	Celador-Lera et al. (2018)
*Azotobacter, Bacillus, Rhizobium, Pseudomonas*	Ammnite A 100	Cleveland biotech, United KIngdom	Plant growth promotion	Odoh et al. (2019)
*Bacillus mucilaginosus, B. subtilis*	CBF	China Bio-Fertilizer AG, China	Plant growth promotion and Biocontrol	Celador-Lera et al. (2018)
*Streptomyces, Nitrobacter, Clostridium, Bacillus, Aerobacter, Achromobacter, Nitrosomonas*	BioPlant	Artemis & Angelio Co. Ltd., Thailand	Plant growth promotion and Biocontrol	Adeleke et al. (2019)
*PGPR consortia*	Bioativo	Embrafros Ltda, Brazil	Plant growth promotion and Biocontrol	Odoh et al. (2019)
*B. amyloliquefaciens, B. megaterium, P. fluorescens*	FZB 24 fl, BactofilA 10	AbiTEP GmbH, Germany	Plant growth promotion and Biocontrol	Odoh et al. (2019)
(*Trichoderma* + *Bacillus amyloliquefaciens* Capsule) (*Rhizobium + Azotobacter* Capsule)	BioCapsules	ICAR- IISR, India / M/s SRT Agro Science Pvt. Ltd, Chhattisgarh	Nutrient mobilization	Saxena et al. (2020)
*Trichoderma harzianum + Bacillus amyloliquefaciens*	Bio-Pulse	ICAR- NBAIM, India	Nutrient mobilization, Biocontrol and Plant growth promotion	Saxena et al. (2020)
Lignocellulolytic fungi (*Phanerochaete chrysosporium + Trichoderma viride + Aspergillus awamori + Pleurotus florida*)	BioCompost	ICAR- NBAIM, India	Soil health enhancement	Saxena et al. (2020)
*Actinobacterial* strains (*Streptomyces viridobrunneus strain + Pan Act1 +* *Streptomyces bullii strain Pan Act2 + Streptomyces* *griseorubens strain Pan Act3*)	Arka Actino-Plus	ICAR-IIHR, India	Plant health management	Saxena et al. (2020)
*Archaea*	Drought alleviating microbial consortium	ICAR- NBAIM, India	Drought alleviation	Saxena et al. (2020)
*Pseudomonas putida P7 + Paenibacillus favisporus strain B30*	CRIDA Mixed Inoculum-I	ICAR- CRIDA, India	Drought stress management	Saxena et al. (2020)
*Pseudomonas putida P45 + Bacillus amyloliquefaciens B17*	CRIDA Mixed Inoculum-II	ICAR- CRIDA, India	Drought stress management	Saxena et al. (2020)
*Bacillus firmus J22*	Drought Guard	ICAR-DGR, India	Drought stress management	Saxena et al. (2020)
*Bacillus firmus J22 + Bacillus subtilis REN51*	SalGuard	ICAR-DGR, India	Salinity stress alleviation	Saxena et al. (2020)
*Pseudomonas* sp. *DSMZ* *13,134*	Proradix	Germany, Itary, Sourcon–Padena	Biocontrol	Saxena et al. (2020)
*Trichoderma viride IIHR Tv-5*	Arka Krishi Veera	ICAR-IIHR, India	Biocontrol	Saxena et al. (2020)
*Pseudomonas* sp. *DSMZ* *13134*	Proradix	Germany, Itary, Sourcon–Padena	Biocontrol	Herman et al. (2008)
*Bacillus* spp.	CIARI-Bioconsortia	ICAR-CIARI, India	Biocontrol	Saxena et al. (2020)
*Bacillus subtilis*	AAU-BioGuard	AAU, Assam, India	Biopesticide	Bora et al. (2021)
*Bacillus subtilis*	Peptilis	IFFCO, India	Biofungicide	IFFCO (2024)
Cyanobacterial strain (*Anabaena laxa and Calothrix elenkinii*)	Cyanobiocon (A & C)	ICAR- IARI, India	Biofungicide	Saxena et al. (2020)
*Pseudomonas fluorescens*	Eco-Pesticide	ICAR- NBAIM, India	Biopesticide	Saxena et al. (2020)
*Pseudomonas taiwanensis*	Arbuscular Mycorrhizal Fungal Inoculum	ICAR-IIHR, India	Biopesticide	Saxena et al. (2020)
*Bacillus thuringiensis (TB160)*	Bentonite-Based Formulation of Bt (TB160)	ICAR-NRRI, India	Biopesticide	Saxena et al. (2020)
*Trichoderma harzianum Th4d*	Trichoderma harzianum Th4d 20 % SC	ICAR-IIOR, India	Biopesticide	Saxena et al. (2020)
*Trichoderma viride*	Bioveer	AAU, Assam, India	Biopesticide	Bora et al. (2021)
*Trichoderma hazarium*	Biozium	AAU, Assam, India	Biopesticide	AAU (2021)
*Metarhizium anisopliae*	Biometa	AAU, Assam, India	Biopesticide	Bora et al. (2021)
*Beauveria bassiana*	Biosona	AAU, Assam, India	Biopesticide	Bora et al. (2021)
*Pseudomonas chlororaphis*	Cedomon	BioAgriAB, Sweden	Biopesticide	Berninger et al. (2018)
*Pseudomonas fluorescens*	Sheathguard	AgriLife, India	Biopesticide	Berninger et al. (2018)
*Agrobacterium radiobacter*	Galltrol‐A	AgBioChem, USA	Biopesticide	Berninger et al. (2018)

The development of biofertilizers has been completely transformed by the identification and isolation of PGPM since the 19th century (Kaminsky et al., 2019; Qiu et al., 2019). Particularly well-researched plant-microbe associations that have been effectively applied in agriculture are arbuscular mycorrhizal associations and rhizobia-legume symbiosis (Bhattacharyya et al., 2016). Both direct (such as nitrogen fixation, phosphorus and potassium solubilization, and phytohormone production) and indirect (including variables such as the synthesis of various compounds like siderophores, antibiotics, hydrogen cyanide, lytic enzymes, and similar bioactive substances) mechanisms that confer resistance to plant pathogens are employed by this microbial inoculant formulation to increase crop yields (Patel et al., 2023). Searching for biofertilizer production has historically focused on screening, characterizing, and creating single isolates with the necessary plant-growth-promoting properties (Gutierrez et al., 2020). In order to develop biofertilizers, the first step is to survey soil from natural ecosystems and select an efficient microbial strain. Next, a collection of microorganisms is separated from their native environment (soil or plant tissue) through in vitro screening for specific taxa that possess one or more traits that promote plant growth (Glick, 2012). Then it is cultivated using specific nutrient medium, scale-up, and formulation using solid or liquid base (Suthar et al., 2017). The strains that prove most effective in stimulating plant development in various environments are then selected for commercialization (Choi et al., 2021). To create the ideal microbial combination for microbial consortia, genomic data and gene expression profiles might be utilized to choose microorganisms with advantageous functional characteristics or metabolic capacity (Toju et al., 2018; de Souza et al., 2020). A number of activities take place before releasing a possible PGPMs inoculant into the market, the majority of which are meant to improve strain survival and efficacy in the field. These activities include characterization, greenhouse and field trials, toxicological profiling, and more. For instance, Inostroza et al. (2017) study demonstrated how microbial consortia from the rhizosphere of *Atriplex sp*. grown in soils of an undisturbed arid Chilean ecosystem without any prior anthropogenic disturbance promoted the growth of wheat seedlings in partially dry soils with low phosphorus content. This demonstrates the potential of dry habitats as a source of microbial inoculants for use in agroecosystems that are nutrient-poor and/or vulnerable to bad weather events. A possible approach to overcoming the challenges of researching natural communities is to design artificial synthetic communities that preserve the salient characteristics of their natural counterparts. Synthetic microbial communities exhibit a defined system behavior with decreased complexity, and they can serve as a model system to evaluate the function of important ecological, structural, and functional aspects of communities in a controlled manner (Suman et al., 2022). The cornerstone of the current top-down and bottom-up approaches to microbial community synthesis is the metabolic relationships between isolates and the functional properties of each particular microbial isolate. According to Grosskopf and Soyer (2014), the fundamental metabolic interactions for the shared substrate or metabolites that result in the establishment of communities include commensalism, competition, predation, cooperation, and amensalism. According to Shayanthan et al. (2022), the synthetic microbial community's method, which combines the ideas of microbial ecology and genetics, has emerged as a promising technique. Either a top-down approach, which focusses on functional definition for a community to characterize its structure and dynamics in detail, or a bottom-up approach, which identifies common interaction patterns and processes among species, can be used to construct synthetic microbial communities. The goal of both approaches is to increase the stability of the microbial community through synergistic interactions among its members. According to de Souza et al. (2016), the synthetic microbial community's technique begins with the separation of microbial cultures from the natural environment and is then developed by modifications of the chosen microbiota to carry out the intended activities for the host plants. Since about 99% of bacteria cannot be cultured, new methods are required to produce a large collection of microorganisms. To find the right medium and culture conditions, one method is to employ metagenomic analysis (Oberhardt et al., 2015). The high-throughput bacterial cultivation techniques like colony picking (Armanhi et al., 2018), cell sorting (Bai et al., 2015), and the limiting dilution method (Zhang et al., 2021) offer viable ways to capture a variety of bacterial species on a large scale (Liu et al., 2019). The efficacy of synthetic microbial communities’ approach can be evaluated both quantitatively and qualitatively using various axenic systems, including FlowPot (autoclaved and washed soil), clay-based (mimic soil), and agar-based (highly artificial and uniformly controlled) systems (Finkel et al., 2019; Zhang et al., 2019) with plant hosts in controlled conditions. Gene expression can also be used to add or remove particular functions, resulting in changes at the functional level (Liu et al., 2019). Moreover, all levels can be used to track the effects of biotic or abiotic disturbances (Melnyk et al., 2019). Lastly, an effective synthetic microbial communities’ with more compatible, effective, and adaptive microorganisms might be evaluated in real-world field settings to counteract the drawbacks of the conventional method (Hart et al., 2017). In order to comprehend plant-microbe interactions utilizing synthetic microbial communities in controlled settings, several research have been carried out in the model plant *A. thaliana* as well as in agricultural crops including maize, soybean, sorghum, and tomato. Bodenhausen et al. (2014) showed that host genotype influences the phyllosphere community composition and abundance significantly using fifty-five *A. thaliana* plant mutants inoculated with synthetic microbial community (7 strains-representatives of the most abundant phyla in the phyllosphere). According to a different synthetic microbial community using 35 strain (34 root associated strains that represent the taxonomic diversity and *E. coli*) investigation, it was reported that root colonization was regulated by microbe-associated molecular patterns (MAMPs) -triggered immunity (Teixeira et al., 2021). As a result, synthetic microbial community are a useful tool for investigating plant-microbe interactions, which are important to take into account when employing microorganisms in extensive agricultural applications. Commercially available PGPMs are used in agricultural ecosystems primarily due to their compatibility and complementarity with certain natural processes pertaining to the nutrient cycle, plant defense against pathogens and parasites, and other related biological processes, according to research on the native microbiome (Adeleke et al., 2019b). In accordance with reports, PGPM applications in the agro-environment are primarily beneficial, benefiting the ecosystem as a whole or in part. The efficiency and repeatability of PGPM-assisted agricultural production techniques can be improved by the employment of microbial consortiums, particularly under challenging environmental circumstances. The inoculant's compatibility with agronomic techniques, like as irrigation and weed control strategies, should also be guaranteed by the formulation process. A designed product is released onto the market so that farmers may use it once it shows promising results in field and greenhouse experiments.

## 6 PGPM in sustaining crop development and productivity

**Fig. 4:**
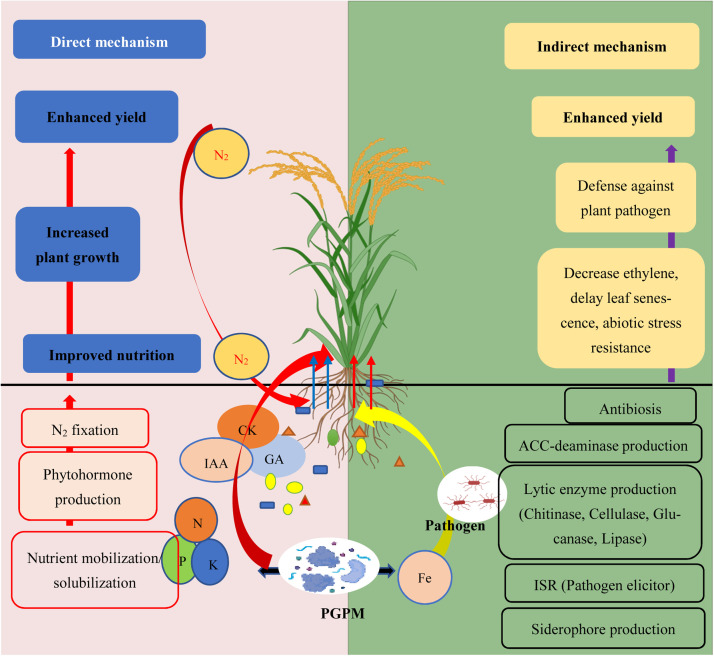
PGPM direct and indirect dual mechanisms in plant growth and health: PGPM may have one or both of these strategies to increase plant productivity and growth. The synthesis of phytohormones, such as auxins, cytokinins, IAA, and gibberellins, and biofertilization, which increases the availability of nutrients by fixing nitrogen and solubilising soil minerals, are direct mechanisms. The production of siderophores (which sequester iron from the soil and supply it to plant cells), root growth stimulation (which promotes root growth), biocontrol (which acts as a biocontrol agent against phytopathogens by producing antibiotics, causing systemic resistance, and competing for nutrients and niches), lytic enzyme production (the production of chitinase, cellulase, glucanase, and lipase), ACC-deaminase and reduce ethylene levels are all indirect mechanisms. Therefore, PGPM encourages the growth of plants' leaf area, chlorophyll content, photosynthetic rates, germination of seeds, vigour of seedlings, height, root development, and biomass output.

For PGPM to be applied in agriculture in a way that supports sustainable crop production, it is important to understand its functions, such as eliminating heavy metals from the soil and offering defense against various forms of drought, heat stress, etc., and help boost a plant's nutrient-absorption capacity, water-use efficiency, and resistance to plant diseases (Armada et al., 2014; Kumar and Verma, 2018). Numerous bacterial taxa that support plant development, resistance to biotic and abiotic challenges, suppression of plant diseases, degradation of xenobiotic chemicals, and favorable effects on yields are found in the plant-associated microbiome (Berg et al., 2014). The development of microbe-based inoculants and formulations has become more demanding due to the breakthroughs achieved in the relationships between plants and microbes in the rhizosphere. According to Johns et al. (2016), these inoculants might be a manufactured mixture of one or more microorganisms or the natural variety found in a rhizosphere. The soil or plant additives known as microbial inoculants can be used to increase crop productivity. Complex and intricate interactions between plant-microbe and microbe-microbe occur during bacterial formulation inoculation in the rhizosphere. These interactions are controlled by the rhizosphere's development of chemical communication. The inoculation-induced signaling cascades in the rhizosphere are actively participated in by the exudation of roots. The establishment of resistance to plant diseases (Bertin et al., 2003), the provision of nutrients to the plants, the promotion of root-root interactions (Mommer et al., 2016a), and the control of resident microbial communities (Sasse et al., 2018) are all made possible by these connections. PGPM improve plant growth and health through direct, indirect, or dual mechanisms (Fig. 4).

By a direct mechanism, microbes improve the uptake of vital nutrients and raise plant hormone levels. Hormone level enhancements have the potential to boost the production of one or more phytochromes, such as auxin, gibberellin, and cytokinin. Using an indirect mechanism, such as the production of the enzyme 1-aminocyclopropane-1-carboxylate (ACC) deaminase, which cleaves the compound ACC, the precursor of ethylene biosynthesis, some of the bacterial microbes reduced the amount of plant hormones (Coutinho et al., 2015). The microbes *Pseudomonas viridiflava*, and *Pseudomonas syringae* reduced the availability of iron to pathogens, generated systemic resistance, and indirectly increased plant growth by producing ecomycins and pseudomycins, respectively (Glick et al., 2015). Therefore, some plants may have a selective advantage over others due to metabolic diversity within the plant microbiome, which has an impact on the ecosystem as a whole (Klironomos, 2002). To coexist with natural microbial communities in the rhizosphere, the imported microbial inoculant faces competition in terms of chemotaxis, root colonization, and nutrient selectivity.

### 6.1 Plant growth and development

For many years, it has been recognized that PGPMs improve plant health and boost production (Conlon et al., 2022; Augusta et al., 2022). Microbial inoculants can be added to soil, seed, leaves, seedling roots, or combination thereof. By improving nutrient availability, regulating phytohormones, and indirectly causing systemic resistance, PGPM directly stimulate plant development (Abhilash et al., 2012; Khan et al., 2020). In soils lacking in nutrients, *Pseudomonas, Bacillus*, and *Mycobacterium* inoculation is frequently more successful in stimulating plant development (Egamberdiyeva, 2007; Mathimaran et al., 2020). In turn, beneficial microbes increase plant tolerance to biotic and abiotic challenges, increase nutrient availability, and regulate phytohormones. All of these processes contribute to improved plant development. According to the previously listed effects, PGPM raises the quantities of ACC-deaminase, auxin, gibberellin, and cytokinin. The microorganisms have the ability to emit volatile metabolites (VOC), which can promote abiotic stress tolerance and disease resistance. PGPM can lessen stress by lowering oxidative stress and raising exopolysaccharides, osmoregulants, and antioxidants (Varma et al., 2019; Khan et al., 2020). Research has been done on PGPM as a potential biofertilizer that might boost macro- and micronutrient availability, encourage plant development, and lessen the requirement for chemical fertilization (Lopes et al., 2021).

As a result, PGPM encourage the growth of roots, plant height, biomass output, germination of seeds, seedling vigor, chlorophyll content, and photosynthetic rates. Given that seed inoculation of PGPM (PSB and *Aspergillus awamori*) has been shown to considerably boost the growth characteristics of mungbean, using microbial compounds may also be a better option (Venkatarao et al., 2017). Increased phosphatases activity in the rhizosphere and *Aspergillus'*s generation of organic acids, which may have converted the native, insoluble phosphate into soluble form. Increased phosphatases activity has been directly linked to plants absorbing more P. Inorganic phosphates that are unavailable can be soluble thanks to increased rhizosphere enzyme activity and the soil medium of *Aspergillus awamori* producing organic acids like lactic and glycolic acids. Since many microorganisms might be at least partially species specific, application of microbial compounds may potentially benefit a larger range of crop species than microbial cells. Plant growth promoters work by secreting a variety of phytohormones into the soil to aid in the growth and development of plants. One example would be (LCOs), which may be used to promote the development of non-leguminous crops and legumes (Chen et al., 2007), both in stressed and non-stressed environments (Babalola and Glick, 2012b; Wang et al., 2016). LCOs encouraged the development of soybean and maize plants (Wang et al., 2015) and increased the production of fruit and flowers in tomato (*Lycopersicon esculentum*) plants (Chen et al., 2007). It has been found that the chemical improves the germination of canola (Schwinghamer et al., 2015) and soybean seeds exposed to high NaCl concentrations (Subramanian et al., 2016). PGPM (*Azospirillum* + PSB) enhance the development of microbiome that, ultimately, increases the microbial activity which help in accumulation of N by rice plant resulting from the increase in SOC (Baishya et al., 2001). This facilitated better crop growth. Application of *Azotobacter* and *Bacillus* sp. helps in growth promotion of maize plants, and forest crops due to their involvement in N fixation (Etesami et al., 2021; Azeem et al., 2022). Jing et al. (2020) reported that the application of genetically modified the root-colonizing strain *Pseudomonas protegens* (Pf-5) which enhanced the production of 2,4-diacetylphloroglucinol and contributed to enhanced antifungal activity, thus helps in promoting the plant growth of wheat and cucumber in nitrogen-deficient conditions effectively. Dasgan et al. (2022) in a study reported the positive effects of three bio-fertilizers, namely bacteria (*Bacillus subtilis, Bacillus megaterium*, and *Pseudomonas fluorescens*), micro-algae (*Chlorella vulgaris* strain) added as nutrient solution; and mycorrhiza (composed of *Glomus intraradices, Glomus aggregatum, Glomus mosseae, Glomus clarum, Glomus monosporus, Glomus deserticola, Glomus brasilianum, Glomus etunicatum*, and *Gigaspora margarita*) applied during seed planting, on basil (*Ocimum basilicum* L.) leaf yield in a floating culture system. The findings show that bacteria produced the highest overall output and leaf area. Mycorrhiza-fed plants had the most leaves and branches, with 94.3 leaves per plant and 24.50 branches per plant, respectively. This biofertilizer was shown to promote the growth of lateral branches in the basil plant without thickening the stems. Thus, PGPM is effective in promoting growth of both soil and soilless system.

### 6.2 Yield and productivity

Ecological and evolutionary processes give rise to plants linked to microbial diversity (Singh et al., 2024; Zilber-Rosenberg and Rosenberg, 2008). One of the PGPMs' common characteristics is that they are a specialized collection of microorganisms that protect and stimulate plants, which in turn provide microbes nourishment. Use of PGPM not only fixes the biological nitrogen but also solubilizes the insoluble phosphates in soil and improves fertilizer-use efficiency (Gogoi et al., 2021). In an experiment conducted by Kumar et al. (2016), the impact of consortia of *Bacillus* sp. (BPR7), *Pseudomonas* sp. (PPR8), and *Rhizobium leguminosarum* (RPN5) significantly boost *P. vulgaris* yield. This might be the consequence of applying *B. subtilis* and *Pseudomonas* sp., which have considerable phosphate-solubilizing activity and enhance yield, along with other phosphate-solubilizers. Microorganisms that mobilize phosphorus efficiently mineralize organic phosphorus compounds and mobilize soluble phosphorus, two forms of phosphorus that are inaccessible (Kirui et al., 2022). For this process, three distinct mechanisms have been identified. In order to mobilize the soluble phosphorus from remote locations that plant roots cannot reach, they first release the phosphatases enzyme, then produce organic acids, and finally engage in symbiotic interactions with other fungal mycorrhiza by absorbing soluble phosphate by hyphae (Etesami et al., 2021). According to Sarmah et al. (2024), black rice (var: Chakhao poireiton) yield and yield-related characteristics are greatly enhanced by using PGPM as a biofertilizer root dip treatment (*i.e*., *Azospirillum* + PSB @ 3.5 kg ha^−1^). The findings show that increased production was obtained from all nutrient sources, including *Azospirillum* and PSB. The root dip treatment's PGPM improved the absorption of macro and micronutrients as well as a significant proportion of nitrogen is fixed by *Azospirillium* when it colonizes the root mass. Root dip application of PSB showed significant higher yields of transplanted rice in acidic soil of North-east India (Kalita et al., 2023). By generating organic acids, citric acids, malic acids, and propionic acids, PSB makes insoluble phosphorus more soluble. These acids boost the availability of phosphorus and help to mobilize it from the soil. The crop's production performance was eventually impacted by the enhanced soil support system and guaranteed nutrient availability provided by the organic inputs taken as a whole. The use of PGPM also helps the crops absorb more nutrients. It might be explained by the use of PGPM (*Azospirillum* and PSB) as a root dip treatment, which may have assisted in the mineralization of soil nutrients and the subsequent build-up of their increased availability in the soil. Maize yield and productivity are greatly enhanced by the application of rhizobacteria (*Rhizobium*) and Arbuscular Mycorrhiza Fungi (*G. intraradices*) (El-Dein et al., 2022). The ability of AMF to provide phosphorus to plants in both organic and inorganic forms is one of its main benefits. Inoculation of *Bradyrhizobium* sp. (isolated from 40 legumes roots and soil samples) on mungbean plant showed a significant increase the yield traits (number of plant pods, total yield and protein%) in seeds compare with non-biofertilized plot. This might be due to the innate ability of the *Bradyrhizobium* sp. in fixing nitrogen, IAA, and siderophores production thus improving the yield of mung bean (Alkurtany et al. 2018). Seeds treated with *Azotobacter chrocoocum, arbuscular mycorrhizal fungi,*AMF and *Bacillus circulans* increase the growth and yield of maize (Gao et al. 2020). Similarly, dual bacterial *inoculum* containing *Azotobacter chroococcum* (MF135558) and *Klebsiella oxytoca* (MF135559) enhances the growth and yield of wheat plants (El-Sawah et al. 2018). PGPM have a positive impact on plant growth and yield as they keep nutrients from leaching out, produce phytohormones, assist plants withstand stress, and aid in iron uptake. According to Mondal et al. (2020), peanut (*Arachis hypogaea* L.) production was increased by *Rhizobium meliloti* innoculation, which was engaged in N_2_ fixation and generated the chitinase enzyme. The microbial community mediates the creation of chitin-catabolic enzymes, or chitinases, which are essential. A chitin-enriched substrate has a greater number of species and microorganisms that promote plant development and are engaged in the metabolism of chitin and nitrogen. In Xinjiang Province, China, Lu et al. (2020) found that inoculating *Vitis vinifera* L. with *P. putida* Rs-198 liquid biofertilizer (Rs198LBF) improved yield. By inhibiting pathogenic microbes, producing growth-stimulating plant hormones, and fostering greater plant disease resistance, a variety of plant-associated *Pseudomonas* improve plant growth and yield. Because of their capacity to produce certain chemicals that are crucial for the synthesis of phosphate solubilizing compounds, siderophores, and N fixation as part of their plant growth-promoting activities, *Pseudomonas* sp. can function as PGPR. Rice grown under elevated CO_2_ by application of PGPM (*Azospirillum lipoferum* strain CRT1, *Azotobactor chroococcum* strain AAU1013, *Bacillus megaterium* strain SZN4, and *Bacillus sporothermodurans* strain CB281428 and *Azolla*) revealed an increase in overall productivity (Bhavya et al., 2023). PGPM increased the photosynthate availability in meristems which causes a rise in the percentage of rapidly growing cells by inducing cell division, and in turn stimulates the development of plants. Auxin production and the cell cycle are stimulated by the non-structural carbohydrates that accumulate in plant tissues under elevated CO_2_, increasing plant productivity overall. Mycorrhiza led to a continuous supply of P as well as few little quantitative of N and K to maize roots, which increase the metabolism, photosynthesis and water uptake resulting in increase in the yield and its components. Following PGPM inoculation in a soil-based media, either alone or in conjunction with mycorrhiza, the biomass and nutrient intake of Sorghum plants increased (Ojuederie and Babalola et al., 2017). Rice yield and yield-related characteristics are considerably improved by bio-inoculation with PGPM (*Trichoderma viride*) (Khadka and Uphoff, 2019). The findings show that *Trichoderma* colonizes roots and sends signals to the plants that cause them to produce growth regulators, build systemic resistance to infections, and increase the amount of nutrients available to the plants by solubilizing P, all of which promote the growth and development of the plants. PGPM (NRICH Bio Organic fertilizer containing mycorrhiza and *trichoderma*) applied four times at 30, 45, 60 and 75 days after transplanting of rice showed positive result for the yield parameters. The beneficial effects of PGPM which enhances soil biological activity and improve nutrient mobilization may be attributed for increased in crop productivity (Noraida and Hisyamuddin, 2021). *Bacillus cereus*, a potassium-solubilizing biofertilizer, enhanced the overall productivity of potatoes by 21 % compared to the untreated plants and improved the health of the potato plants (li et al. 2021). Similarly, in another 2-year research trial, application of potash solubilizing bacteria (*Bacillus proteolyticus + Serratia liquifaciens*) @ 3.5 kg as seedling root dip treatment in rice showed substantial enhancements in both quality and overall production (Dambale et al., 2024). Siderophore synthesis, complexation, and exchange reactions are aided by potassium-solubilizing biofertilizers (Sattar et al., 2019). Crop growth and productivity are increased by its precise control of stomatal closure and opening, nutrient absorption, protein synthesis, and product quality. It also confers resistance to environmental stressors (Santosh et al., 2022). PGPR and other free-living endophytic bacteria have received the majority of research focus to far. However, beneficial fungi that are free-living and known as PGPF could be just as important.

## 7 Plant growth-promoting microorganism (PGPM) in sustaining soil health

Given the expense and environmental impact of synthetic fertilizers, stress-tolerant plant growth-promoting organisms may be a more affordable and sustainable method of recovering land that has been abandoned for agricultural purposes. Since nutrients can increase a plant's resistance to abiotic stress, improving soil fertility will inevitably be important as more land needs to be recovered for crop production (Naamala and Smith, 2020). As a result, more environmentally friendly methods of solving the problem are required. Microbial inoculants are a viable method for increasing soil fertility because there aren't many other options and research has shown promise in this area. Growing plants that alter the microbial diversity of soil, introducing new microbial species to the rhizosphere, and adjusting environmental factors like temperature, pH, and moisture are some of the ways it may be aided (Finkel et al., 2017; Pineda et al., 2017). Colonization suggests that a tiny amount of or short-term life activities of the applied biofertilizer may influence the species or proportion of dominating bacteria in the soil ecosystem (Zhao et al., 2023). The role of microbes is crucial, because they are responsible for organic matter decomposition, nutrient cycling, soil fertility and quality for which maintaining high levels of microbial diversity in soil is the key for sustainable agriculture and supply a wide range of ecosystem services which is crucial for human well-being and sustainable quality of life (Gogoi et al., 2021). It also provided more labile carbon substances needed for maintenance of a larger population of soil bacteria and fungi and is highly potent to augment microbial and enzymatic activities in maintaining soil health and improve soil ecosystem services. Beneficial microorganisms have often been recommended for sustaining soil fertility and soil health but also increase agronomic efficiency, for which it has been proposed as an alternative efficient practice for sustainable crop production. PGPM contribute to soil aggregation, which improves soil structure and aeration (Bashan et al., 2013). This aid root growth, allowing better access to nutrients and water. Additionally, microbial inoculants have the potential to improve soil structure and biodiversity (Berg and Koskella, 2018). Using biofertilizers containing *B. amyloliquefaciens* can successfully reduce the number of harmful bacteria. This results in an increase in bacterial abundance and a decrease in fungal abundance (Fu et al., 2016). Biofertilizers have also been shown to modify the soil microbiome in sugarcane, lucerne, apricot, and other crops (Baldi et al., 2021; Liu et al., 2022; Zhao et al., 2022). PGPM has the potential to increase the availability of phosphorus in the soil for plant uptake by solubilizing/mobilizing inorganic phosphorus. According to Goldstein and Krishnaraj (2007), phosphate solubilizing microorganisms are those that, in the native soil ecosystem of the microorganism, transform sparing soluble organic or mineral P into soluble orthophosphate in a way that greatly increases P availability to a particular plant or plant population. The increased efficacy of microbial consortiums under harsher environmental conditions—more precisely, in a tomato production system in Israel's Negev desert with mineral fertilization at a high pH (7.9), low fertility, and sandy soil—was an especially intriguing discovery (Mahmood et al., 2019). Consortia inoculation was linked to specific alterations in the bacterial community's rhizosphere structure, particularly in relation to Sphingobacteria and Flavobacteria, which are recognized to be defenders against drought stress. Using thermogravimetry-differential thermal analysis (TG-DTA) and NIR spectroscopy, Baldi et al. (2021) observed a significant change in the chemical decomposition over time and more rapid degradation of organic matter when *Trichoderma* sp. was applied to an apricot orchard in Italy. This study provides additional evidence of how PGPMs application can modify the soil microbiome.

### 7.1 Soil physical and chemical properties

Utilized as biofertilizers, PGPMs increase the availability of nutrients by solubilizing soil minerals including potassium and phosphorus and nitrogen fixing atmospheric N, thus improving the overall soil health in the long term. Tisdall and Oades (1979) reported that soil organic matter (SOM), which is generated by AMF, promotes soil fertility and health by facilitating microbial colonization and soil aggregation. Applying PGPMs to soil can enhance its physical quality, particularly in terms of holding onto moisture because of increased microporosity (Dhawi et al., 2023). Compaction and soil erosion can be successfully decreased by increasing the stability of soil aggregates (Erktan et al., 2015). By linking soil particles with their extracellular polysaccharides, microorganisms—particularly fungi fungi and actinomycetes—play a crucial role in the formation of stable soil aggregates (Pokharel et al., 2013). Extracellular polysaccharides and lipids are also produced during the microbial breakdown of new organic matter, and these substances aid in the development and stability of soil aggregate structure (Wei et al., 2024). *B. thuringiensis* alters the carbon-nitrogen ratio by mineralizing organic matter, which impacts the protists and microbiota structure in the soil (Goldstein and Krishnaraj, 2007). Certain PGPM are also implicated in soil aggregation (Sandhya et al., 2009). AMF contribute to soil texture and aggregation as well as the balance of C, P, and N through nutrient recycling (Correa et al., 2015). The bulk density of the normally-occurring soil was considerably stabilized by PGPM (*Azospirillum/Azotobacter* + PSB). Better aggregation and greater soil porosity, which eventually boosted the soil's capacity to store water, may be the cause of the notable fall in bulk density (Baishya et al., 2001). He also reported that application of PGPM resulted in a notable improvement in nutrient levels of the soil. N_2_ fixation is aided by *Azospirillum/Azotobacter*, while plants' availability of P is increased by PSB. An increase in DTPA micronutrients may be the result of the advantageous properties of organics, which aid in the provision of micronutrients in an easily obtainable form. The addition of PGPM (*Bacillus subtilis* and *Bacillus megaterium*) reduced the effects of salt stress, enhanced water-salt dispersion, decreased plant Na+ absorption, and considerably decreased soil specific gravity during the course of a two-year field trial (Ding et al., 2024). Microorganisms' metabolic processes during the breakdown of organic matter result in the production of extracellular polymers and sticky molecules that help soil particles bind together, forming aggregates and improving soil particle cohesion. In a study conducted by Li et al. (2023), AMF strain (*Funneliformis mosseae, Laroideoglomus etunicatum*, and *Rhizophagus intraradices*) inoculation contributed encourage their host plants to secrete organic acids like citric and malic acid through chelation, thus raising the pH of the soil. In alkaline soils, AMF can also encourage the release of organic acids like valeric acid, which speeds up the breakdown of polycyclic aromatic hydrocarbons and lowers soil pH. *B. subtilis* secretes organic acids that can improve the pH of the surrounding soil and encourage plant growth in saline-alkali land (Alakhdar et al., 2020). The solubility of various metal ions, nutritional availability, and physical characteristics of soil are all dependent on the pH of the soil (Dutta and Bora, 2019; Msimbira and Smith, 2020). According to Bakhshandeh et al. (2019) *Bacillus* and *Trichoderma* in *Glycine* max and *Rhizobium* and *Paenibacillus* increasing pH tolerance in *Triticum*, plant beneficial microbe inoculation is a viable technique to improve plant tolerance to pH extremes (El-Sayed and Hagab, 2020). Under salt and aluminum toxicity, *pseudomonas* enhances development and increases resistance to high salinity conditions in *Zea mays* (Samaddar et al., 2019) and *Capsicum annuum* (Zerrouk et al., 2019). PGPMs consortium (*Bacillus megaterium* BHUPSB14, *Pseudomonas fluorescens* BHUPSB06, *Pseudomonas aeruginosa* BHUPSB01, *Pseudomonas putida* BHUPSB0, *Paenibacillus polymixa* BHUPSB17, and *Trichoderma horzianum*) application also improved the soil pH and EC and soil nutrient status (OC, N, P and K) in drought stress condition (Krishna et al., 2024). Aechra et al. (2022) in a 2-year research trial reported that seed inoculation with biofertilizers (*Azotobacter +* Phosphorus solubilizing bacteria + Potash mobilizing bacteria + Zinc solubilizing bacteria) improved the pH and EC. Water holding capacity, cation exchange capacity, organic carbon, N, P, K, Zn, Fe, Mn and Cu was also significantly affected by application of PGPM. In a two-year study on a rice-linseed relay cropping system with seedling rood dip treatment with *Azospirillium* and PSB, Sarmah et al. (2024) found that the soil's N, P, K, and OC chemical characteristics significantly improved. Microbial inoculants serve as conduits to improve the availability and uptake of essential plant nutrients, including potassium, zinc, iron, phosphorus, and nitrogen (Nam et al., 2015; Gupta et al., 2015). If certain minerals are not present or are not easily available, they may limit plant growth. Dutta et al. (2024a) reported that PGPMs [*Azospirillum amazonense* + *Bacillus megaterium* @ 4 kg ha^−1^, PSB as + KSB (*Bacillus proteolyticus + Serratia liquifaciens*) @ 4 kg ha^−1^ as seedling root dip] showed significant improvement on soil N, P and K content. The advantages of inoculation are indisputable; PGPM application enhances the physical and chemical characteristics of the soil through a different mechanism of action, and promotes and increases plant resistance and growth by releasing phytohormones or small molecules and volatile compounds (Krishna et al., 2024).

### 7.2 Soil microbial and enzymatic activity

AMF strain (*Funneliformis mosseae, Laroideoglomus etunicatum*, and *Rhizophagus intraradices*) inoculation contributed in the mycorrhizal rhizosphere, high activities of oxidoreductases such as polyphenol oxidase, dehydrogenase, dioxygenase, catalase, and dioxygenase have been observed (Li et al. 2023). The research further clarified the changes in rhizosphere substances induced by AMF such as organic acids including arachidonic acid, octadecanedioic acid, α-linolenic acid, 10,12,14-octadecarachidonic acid and 5-methoxysalicylic acid that can act as co-metabolic substrates for certain microbial species to metabolize PAHs were significantly increased in AMF-inoculated treatments. In drought stress conditions the soil biological (bacteria, fungi and *Actinomycetes* population) and enzyme (dehydrogenase, phosphatase, and urease activities) were found to be improved by application of PGPMs consortium (*Bacillus* sp. + *Pseudomonas sp*. + *Paenibacillus* sp. and *Trichoderma* sp.) (Krishna et al. 2024). Plant and soil health can be impacted by microbes living in rhizosphere or soil areas. The lack of SOM in the soil is an issue that PGPM can solve (Trabelsi and Mhamdi, 2013). According to Singh et al. (2019), SOM in soil controls a number of parameters, including soil conservation and restoration, micro- and macronutrients, soil porosity, permeability, ammonium ions (NH_4_+), and nitrogen trans-formation. It also balances the carbon to nitrogen ratio. Dutta et al. (2024b) also reported that PGPMs [*Azospirillum amazonense* + *Bacillus megaterium* @ 4 kg ha^−1^, PSB as + KSB (*Bacillus proteolyticus + Serratia liquifaciens*) @ 4 kg ha^−1^ as seedling root dip] showed significant improvement on SMBC in acidic soil of Assam. The addition of PGPMs (*Bacillus subtilis* and *Bacillus megaterium*) increased the soil microbial biomass (C and N) and markedly elevated the urease and sucrase activities in saline soil (Ding et al., 2024). Microbial activity, metabolism, and nutrient conversion are frequently correlated with the level of soil enzyme activity, which is intimately related to the microorganisms' breakdown of SOM (Sinsabaugh et al., 2009). *Bacillus subtilis* breaks down organic materials and releases nutrients, which might affect soil urease and invertase activity in biofertilizers (Akinsemolu et al., 2024). The spores of *Bacillus subtilis*, a common PGPM found in the inter-root zone, are long-lived and weather-resistant, allowing them to survive in saline soil environments and aiding in the decomposition of SOM and the transformation of nutrients (Mahapatra et al., 2022). In order to determine the impact of PGPM on the population of soil microbes and the activity of enzymes under rice crops, Phukan et al. (2020) carried out an experiment. According to her, using PGPMs (*Bacillus* and *Pseudomonas* sp.) greatly raised the number of bacteria and enzyme activity. When *Bacillus cereus* and *Pseudomonas fluorescens* were injected, the fungal population was found to be much larger than when no inoculation was done. This might be releases of phytohormones, which draw microbes to the rhizosphere and encourage microbial activity. When *Pseudomonas fluorescens* and *Bacillus cereus* were added to the mixture, the phosphomonoesterases and dehydrogenase activity was much greater than when no inoculation was made. Increased microbial population may result in increased microbial activity in the soil, which would then improve the soil's enzyme content. According to Gogoi et al. (2021), in a long-term rice-rice environment, PGPM (*Azolla* as biofertilizer) greatly enhanced soil microbial and enzymatic activity and their contribution to soil ecosystem services (SES). It's possible that PGPM supplied more labile carbon molecules that were required to support a higher number of fungus and bacteria in the soil. Because Azolla, increased the number of bacteria in the soil, which may be the cause of the highest number of bacterial colonies in the soil. Due to increased microbial activity and the consequent release of more organically bound P in the soil, the phosphomonoesterase (PMEase) activity in the soil beneath the rice-rice system was much higher in the PGPM supplemented treatments. Additionally, the continuous PGPM supplementations that raised the SOC content also had the greatest levels of fluorescein diacetate (FDA), which may have boosted the activities of lipases, esterases, and proteases in the soil. These enzymes in the soil hydrolyze the FDA, resulting in a broad microbial action. In the end, PGPM boost the growth of microflora, which raises soil health globally and preserves soil ecosystem services. An experiment was carried out by Nath et al. (2012) showed that, PGPM increases the enzymatic and microbial activity of soil. When inorganic fertilizers were significantly reduced and PGPM, used, FDA hydrolysis increased. Since the presence of organic substrate stimulates the production of the enzyme, increased PMEase activity may be the result of more P being released from its organic bond. In summary, he concluded that PGPM have the ability to increase soil enzyme activity and boost the microbial biomass carbon and organic carbon of soil.

### 7.3 Soil bioremediation

Pollutants found in the environment, including pesticides, heavy metals, hazardous waste, polycyclic aromatic hydrocarbons (PAH) are caused by human activity and pose a serious threat to soil fertility. According to Li et al. (2023), inoculation of AMF strains (*Funneliformis mosseae, Laroideoglomus etunicatum*, and *Rhizophagus intraradices*) significantly increased the levels of polyphenol oxidase, laccase, and dehydrogenase, which are essential for the biodegradation of PAHs. The results have validated the critical roles of organic acids and soil enzymes in plant-AMF combined remediation of PAHs, and they offer an efficient method for employing AMF to clean up PAH-polluted soils. The majority of microbes are toxic to heavy metal pollution in soils, and it can also reduce the potency of inoculants. According to Mimmo et al. (2018), heavy metals decrease soil fertility, have an impact on the rhizosphere microbial population, decrease plant photosynthetic efficiency, create nutritional imbalances, and lower yields. Eco-friendly bioremediation has drawn a lot of interest due to the inefficiency of traditional physicochemical approaches. They can improve phytoremediation processes because of their many known positive effects, which include modifying metal phytoavailability by releasing chelating agents, acidification, phosphate solubilization, and redox changes (Veerapagu et al., 2023). Thus, in addition to phytoremediation, the use of PGPMs provides a novel method of removing pollutants from the environment. Microbe-based bioremediation is a long-term, sustainable method of repairing damaged environments since it is both economical and successful in returning the environment to its original form. Microbes can detoxify heavy metals naturally, through genetic modification, or by adding native microbial strains (Rashid et al., 2019). Plant tolerance to metals can be enhanced by metallothioneins generated by PGPM (e.g., *P. putida* and *Mycobacterium tuberculosis*), as certain gene transformation studies have persuasively shown (Nanda et al., 2019). *E. Coli* cells expressing SUMO-*Sh*MT3 bioaccumulated Cd^2+^, Cu^2+^, and Zn^2+^, as demonstrated by Li et al. (2021). According to Kumari and Das (2019), the biofilm-forming marine bacterium *P. aeruginosa* N6P6 with the *bmtA* gene was resistant to a number of metals, including Pb, Cd, Hg, Cr, and Zn. Seed priming biosurfactant producing *Pseudomonas* sp. AJ15 on *Withania somnifera* under petroleum toxicity showed positive results on detoxification of petroleum contaminated soils for growing economically important crops. The petroleum degrading biosurfactant degrade and utilized petroleum as a carbon source (Das and Kumar, 2016). Similarly, *Glomus intraradices* and *Acinetobacter* sp. application in oats showed positive effects on bioremediation of petroleum contaminated soil (Xun et al., 2015). The accumulation of superoxide dismutase, catalase and peroxidase, decreased malondialdehyde (MDA) and free proline contents, increasing soil enzymes urease, sucrase, and dehydrogenase, and elevated the tolerance to hydrocarbon contaminants (Chitara et al., 2021). Microorganisms use a variety of techniques, such as biosorption, adsorption, metal binding, vacuolar compartmentalization, extracellular mobilization, or immobilization of metal ions, to lower the active concentration of metal ions present in contaminated environments (Ray et al., 2020). Beneficial microbes including *Pseudomonas aeruginosa, Alcaligenes feacalis,* and *Bacillus subtilis* are a successful remediation approach in polluted soils, boosting plant tolerance to heavy metals (Ndeddy-Aka and Babalola (2016)). Amendments containing organic matter can affect soil microbes. Soil organic matter is increased and soil fertility is enhanced by adding PGPM, such as microbial inoculant, biofertilizer, etc. It makes use of the biological processes that bacteria and plants have to eliminate dangerous contaminants and return the environment to its natural state. The important components needed to remediate industrial wastes like heavy metals, pesticides, and toxic chemical fertilizers are microorganisms (bacteria, yeast, fungi, and even archaeon) and plants (Kothe, 2009; Verma and Jaiswal, 2016). Hassen et al. (2018) in a study revealed that application of *Pseudomonas rhizophila* S211 on *Artichoke* reduced the pesticide residue accumulation in soil. *Pseudomonas rhizophila* key genes aid in the synthesis of ACC de aminase, putative dioxygenases, auxin, pyroverdin, exopolysaccharide levan and rhamnolipid biosurfactant allows *Artichoke* plant to utilize various kinds of pollutants to grow in polluted soil. *Pseudomonas* sp. and *Bacillus* sp. in rice utilize glyphosate (herbicide) as a phosphorus source and aid in its degradation (Wijekoon and Yapa, 2018). Trifi et al. (2020) found that *Pantoea* sp. BRM17 inoculation on Canola (*Brassica napus*) effectively removed the phosphogypsum (a by-product of the phosphate fertilizer industry) through mechanism of siderophores transportation, IAA, exopolysaccharides, ammonia, and ACC deaminase activity. Many plants have been examined to see whether they can absorb large amounts of heavy metals. There are several different PGPM that have been shown to effectively support phytoremediation (Glick, 2010). Jing et al. (2014), for instance, extracted metal-resistant PGPM from *Polygonum pubescens* cultivated in soil contaminated with metals. After being injected into *Brassica napus* for the purpose of accumulating heavy metals, the isolated strains of *Enterobacter* sp. and *Klebsiella* sp. were identified. These bacteria enhanced plant growth and *B. napus* uptake of Cd, Pb, and Zn. Using a variety of chemicals and root exudates, the plant interacts with microorganisms throughout growth to promote bacterial survival and activity and improve the containment, transformation, or degradation of pollutants (Kuiper et al., 2004). These compounds have the capacity to increase pollutants' phytoavailability (Abhilash et al., 2012), mobility (Ojuederie and Babalola, 2017), and chelating agent activity. Chelation is presumably the primary process by which bacteria carry out bioremediation. To take up metal ions and stop them from being resorpted, plants and microorganisms can produce organic chelating substances such as siderophores, organic acid anions, metal-binding compounds, and biosurfactants (Gadd, 2004; Sessitsch et al., 2013). Wu et al. (2018) observed that the inoculation of *Buttiauxella* sp. SaSR13 led to a considerable increase in the bioavailability and plant absorption of Cd by increasing the concentration of root exudates of *S. alfredii*, particularly malic and oxalic acids. By solubilizing and micellizing hydrophobic pollutants, biosurfactants can also facilitate their entrance into the aqueous phase (Wang et al., 2022). These micelles and dissolved pollutants facilitate the removal of metals by soil washing or facilitate their easy absorption by plants (Akbari et al., 2018). Thus, the presence of microorganisms that produce biosurfactants in polluted soils can effectively increase the mobility of metals (Lal et al., 2018). According to San Martín et al. (2021), *Pseudomonas* Y3-B1A, which produces rhamnolipids, has a maximum vanadium removal effectiveness of 85.5 %. *Microbacterium* sp. EIKU5, *Shinella* sp. EIKU6, and *Micrococcus* sp. EIKU8 showed resistance to metals and oxidized the Arsenic (As) and uranium (U) (Bhakat et al., 2019). This is because they function as biological catalysts in a bioremediation system (van Dillewijn et al., 2009), which is set up by components that are appropriate to fix contaminated environments, such as plants that are suitable to remove and restrain metals from the ground and microorganisms that can take up and transform heavy metals. Even though heavy metals seem like difficult to manage contaminants, bioremediation is a desirable way to treat soil that has been contaminated by them; moreover, using both microbes and plants together is a way to guarantee a more thorough remediation (Chibuike and Obiora, 2014). Since the specific processes of plant and microbe bioremediation are well established, a standardized combination of them may be used either in situ or ex situ bioremediation.

## 8 Frontiers of PGPM as microbial inoculants/biofertilizers (current and future)

For the past several decades, the marketing of microbial inoculants has been used in particular to reduce the use of chemical fertilizers to some extent (Paudyal and Gupta, 2018). Erosion, organic carbon loss, nutrient depletion, soil sealing, climate change, and other factors are causing the agro-ecosystems to deteriorate more quickly globally, which is leading to the loss of those potential PGPM genera (FAO, 2015). Microbial culture collections (MCC) play a critical role in accomplishing this objective as the preservation of this biological variety is necessary for its reintegration into agro-ecosystems (Valenzuela-Ruiz et al., 2018). Accordingly, the need to preserve these microorganisms as well as research them for the creation of new agro-biotechnologies (de los Santos-Villalobos et al., 2018) is exacerbated by the rise of biological concerns, global food insecurity, and the ongoing discovery of new microbial species or subspecies (de los Santos Villalobos et al., 2019). Three steps must be connected in order for the anticipated benefits of preserved PGPM in MCC to contribute to global food security: (i) isolating microbial strains in agro-ecosystems; (ii) preserving these strains in an MCC; and (iii) bioprospecting these promising strains in order to transfer and create sustainable agro-biotechnological alternatives. This is necessary for the successful widespread use of microbial inoculants. Therefore, digitizing, making easily accessible, and disseminating biological information for every conserved microbial strain—including metabolic and genetic diversity—is crucial to resolving bottlenecks in MCC's operations. A growing body of research is being done on the use of microbial products as agricultural inputs and biofertilizers nowadays. Nevertheless, PGPMs full potential as biofertilizers remains mostly unrealized. The commercialization of biofertilizer and microbial inoculants is still very low worldwide. In the 21st century, microbial inoculants/biofertilizers are emerging as a viable substitute for sustainable crop production (Allouzi et al., 2022; Nosheen et al., 1868). They have also been suggested as a way to improve plant resilience and the rhizosphere's ability to withstand biotic and abiotic challenges (Ibáñez et al., 2023). The important element influencing the efficacy of microbial inoculants in the field is the climate. Global estimates indicate that abiotic factors account for 50 % of crop yield losses, with temperature variations accounting for the majority (27 %). Other factors that impact crop yields and the effectiveness of microbial inoculants include salinity (10 %), drought (9 %), and other types of stress (4 %) (Ibarra-Villarreal et al., 2021). The selection and isolation of effective PGPM has been aided by global research advancements on the diversity, roles, and potentials of the native microorganisms. The rising demand for organic food items has had a significant impact on the global growth in demand for biofertilizers. Legume and N_2_-fixing inoculants already hold a significant market share in the global biofertilizer industry (Vassilev et al., 2015). The worldwide biofertilizer industry is currently dominated by rhizobial inoculants, with PSB and other bioinoculants accounting for less than 30 % of the market (Transparency Market Research, 2022). Presently, a number of microbial genera are utilized in the creation of microbial inoculants because of their metabolic diversity; for example, numerous *Bacillus* sp. (Ibarra-Villarreal et al., 2021 and *Klebsiella* sp. (Díaz-Rodríguez et al., 2021). However, to address soil nutrient shortages, P, K, and Zn-based biofertilizers are increasingly evolving into substantial bioinoculants, and potassium solubilizing microbes are now widely utilized as inoculants in several countries with croplands low in K (Teotia et al., 2016). India is purportedly the fourth-biggest global user of K bioinoculants, with Brazil, the USA, and China leading the pack in terms of total use of these microbial products (Matich, 2016). At a compound annual growth rate (CAGR) of 12.04 %, the global market for biofertilizers used in crop production is expected to increase from US$2.02 billion in 2022 to US$4.47 billion by 2029 (Fortune Business Insights, 2022). Nevertheless, given the positive economic outlook, a number of methodological, environmental, and regulatory issues are impeding their progress. International regulatory frameworks need to be redefined, improved based on emerging technology, and cross-sector interdisciplinary collaboration encouraged in order to promote biofertilizers as economical and environmentally benign substitutes for chemical fertilizers.

In relation to the potential for agro-biotechnological applications of PGPM, microbial communities inside agro-ecosystems have gained increased significance in recent times for the creation of novel microbial inoculants. Successful isolation, identification, and characterization of potential PGPM are essential for the widespread use of microbial inoculants (Díaz-Rodríguez et al., 2021). Although PGPM has been used more widely in agricultural production as a novel approach to reduce environmental issues, its relevance in the agroecosystem has been disregarded. The function of PGPM in agro-ecosystems may be strengthened in the near future by contemporary technology, such as bioengineering instruments, to offer long-term solutions to a range of environmental issues. Appropriate legislative and regulatory frameworks will need to replace the current ones, which are too restrictive and prevent the widespread use of biofertilizers. It will eventually be possible to loosen the strict regulatory frameworks and allow the widespread adoption of these microbial resources when it is acknowledged that a specific legislative framework is required for biofertilizers and the implementation of alternative crop fertilization mechanisms to promote the development of sustainable agricultural technologies. In terms of comprehensive legislative frameworks pertaining to biofertilizers, India and China rank highest globally, with the United States following suit (Raimi et al., 2020). Under Section 3 of the Essential Commodities Act, 1955 and the Fertiliser (Control) Order, 1985 (Praveen and Singh, 2019), the Indian government has officially designated "biofertilizer" and established appropriate regulatory frameworks. Eight criteria have been established by China as the legal quality standards for biofertilizers: the quantity of live cells, the carbon and water content, the pH, the granule size (for solid goods), the appearance, contamination, and validity (Suh et al., 2006). The USDA National Organic Programme (NOP) regulates the use of biofertilizers in organic farming, whereas the Environmental Protection Agency (EPA) is in charge of biopesticides in the United States, which include both microbial pesticides and plant-incorporated protectants (Malusá and Vassilev, 2014). While many of the current biofertilizers are mostly made of naturally occurring rhizobacterial strains selected for their ability to stimulate plant growth, it is still necessary to produce genetically engineered inoculants that are likely to be more effective at stimulating plant growth. The largest challenge will still be for scientists to persuade the public and international regulatory bodies that these genetically modified creatures are safe. The novel biofertilizers are being screened, characterized, and used in an effort to gain industrial-scale adoption. This is being spurred by developing technologies like as encapsulation and metaomics. But it appears that the relationship between farmers, legislators, business, and laboratory research will be the crucial factor in promoting biofertilizers as a more environmentally friendly tool. As a practical substitute for the chemical methods, the culmination of these many contributions ought to be realized in optimized, economically viable bioformulations.

The new innovative methods will be needed to understand the interactions between plants and biofertilizer inoculants. Future studies should also carefully choose the bacteria of the rhizosphere and evaluate them in-situ before using them as plant inoculants. In order to create appropriate bioformulations for certain soils and crops, for instance, multi-omics techniques can be extremely helpful in our understanding of complicated plant-microbial symbioses (Kaul et al., 2016; López-Mondéjar et al., 2017; Ding et al., 2021; Yamazaki et al., 2021). In order to enable their implementation, these unique techniques will eventually improve the full characterization of PGPM and their impact on plant nutrient absorption and other plant growth promoting characteristics. Research on these should thus be given priority. Eventually, it will be critical to recognize the obstacles to the development and use of biofertilizers as well as the solutions for these issues. For example, crop species, soil complexity, and climate all affect how effective biofertilizers are in the field. In the future, the investigation of appropriate biofertilizers ought to be driven by agronomists who comprehend the relationship between crops, climate, and nutrients around the globe. The advancement of screening methods, such as the measurement of antioxidant enzymes and other substances that can aid in plant development, must be a part of the future prospects of microbial applications. Better inoculation techniques, a description of the rhizosphere microbiome of each species under investigation, and an examination of the relationship between plants and PGPM are all necessary for future research. It is anticipated that software will be developed in the future to show the optimum inoculation technique, the optimal PGPM to help a particular plant species, and how it acts under various abiotic conditions (Díaz-Rodríguez et al., 2021). As a consequence, microorganisms will be chosen more effectively, which will boost plant development and promote environmentally friendly and sustainable agriculture. And in order to manipulate native PGPM with the right genes for improved expression of biofertilization activities for field applications, genomic engineering may be required and, certain additions may enhance the shelf life, field effectiveness, and stability of the product.

## 9 Conclusion

This review paper aims to provide a comprehensive understanding of plant-microbe interactions and their significance in sustaining crop productivity and soil fertility. The underlying mechanisms, exploring influencing factors, and discussing practical implications and future directions, it seeks to inform researchers for advancing sustainable agricultural practices. Promising approaches for ecologically sustainable farming may result from beneficial microbial-plant interactions, in sustainable agriculture, the development of biofertilizer, biocontrol, and bioremediation agents has been greatly aided by the interaction between plants and microbes. And to improving plant nutrition and production, PGPM is crucial for maintaining ecological stability, an enhancing plant health, a variety of interacting microorganisms shield plants from biotic and abiotic stress. It is clear from this that the interacting microbes work to maximize a variety of biological processes occurring in the soil in order to provide a thriving, healthy environment that guarantees the crop will receive enough nutrients. Nonetheless, public education on PGPM's application in agriculture and its wider use is imperative.

## CRediT authorship contribution statement

Bibek Laishram: Writing – review & editing, Writing – original draft, Visualization. Okram Ricky Devi: Writing – review & editing, Writing – original draft. Rinjumoni Dutta: Writing – review & editing. T. Senthilkumar: – review & editing, Writing- Visualization. Girish Goyal: review & editing, Writing. Dinesh Kumar Paliwal: review & editing, Writing. Narinder Panotra: review & editing, Writing. Akhtar Rasool: Supervision, validation, Conceptualization and editing.

## Funding

No Funds received from any organization.

## Declaration of competing interest

The authors declare the following financial interests/personal relationships which may be considered as potential competing interests.

## Data Availability

No data was used for the research described in the article.
